# Evaluation of partial volume correction and analysis of longitudinal [^18^F]GTP1 tau PET imaging in Alzheimer's disease using linear mixed-effects models

**DOI:** 10.3389/fnimg.2024.1355402

**Published:** 2024-03-28

**Authors:** Sandra M. Sanabria Bohórquez, Suzanne Baker, Paul T. Manser, Matteo Tonietto, Christopher Galli, Kristin R. Wildsmith, Yixuan Zou, Geoffrey A. Kerchner, Robby Weimer, Edmond Teng

**Affiliations:** ^1^Clinical Imaging Group, Genentech, Inc., South San Francisco, CA, United States; ^2^Molecular Biophysics and Integrated Bioimaging, Lawrence Berkeley National Laboratory, Berkeley, CA, United States; ^3^Biostatistics, Genentech, Inc., South San Francisco, CA, United States; ^4^Roche Pharma Research and Early Development, Roche Innovation Center Basel, F. Hoffmann-La Roche Ltd., Basel, Switzerland; ^5^Biomarker Development, Genentech, Inc., South San Francisco, CA, United States; ^6^Data and Statistical Science, Product Development, Hoffmann-La Roche Ltd., Basel, Switzerland; ^7^Translational Imaging, Genentech, Inc., South San Francisco, CA, United States; ^8^Early Clinical Development, Genentech, Inc., South San Francisco, CA, United States

**Keywords:** tau PET, Alzheimer's disease, longitudinal change, linear mixed-effects models, neuroimaging

## Abstract

**Purpose:**

We evaluated the impact of partial volume correction (PVC) methods on the quantification of longitudinal [^18^F]GTP1 tau positron-emission tomography (PET) in Alzheimer's disease and the suitability of describing the tau pathology burden temporal trajectories using linear mixed-effects models (LMEM).

**Methods:**

We applied van Cittert iterative deconvolution (VC), 2-compartment, and 3-compartment, and the geometric transfer matrix plus region-based voxelwise methods to data acquired in an Alzheimer's disease natural history study over 18 months at a single imaging site. We determined the optimal PVC method by comparing the standardized uptake value ratio change (%ΔSUVR) between diagnostic and tau burden–level groups and the longitudinal repeatability derived from the LMEM. The performance of LMEM analysis for calculating %ΔSUVR was evaluated in a natural history study and in a multisite clinical trial of semorinemab in prodromal to mild Alzheimer's disease by comparing results to traditional per-visit estimates.

**Results:**

The VC, 2-compartment, and 3-compartment PVC methods had similar performance, whereas region-based voxelwise overcorrected regions with a higher tau burden. The lowest within-subject variability and acceptable group separation scores were observed without PVC. The LMEM-derived %ΔSUVR values were similar to the per-visit estimates with lower variability.

**Conclusion:**

The results indicate that the tested PVC methods do not offer a clear advantage or improvement over non-PVC images for the quantification of longitudinal [^18^F]GTP1 PET data. LMEM offers a robust framework for the longitudinal tau PET quantification with low longitudinal test–retest variability.

**Clinical trial registration:**

NCT02640092 and NCT03289143.

## 1 Introduction

The evaluation of the longitudinal change in tau burden in patients with Alzheimer's disease (AD) via tau positron emission tomography (PET) is crucial for understanding the pathological progression and potential response to intervention in clinical trials. The observation periods in interventional phase 2 clinical trials tend to be relatively short (1.5–2 years), and the change in the tau PET signal over such intervals is relatively small. Although direct comparisons of the published data are limited by differences in tau PET ligands, study populations, target regions of interest (ROIs), and image analysis pipelines, a range of rates of longitudinal change have been reported. Teng et al. (2022) observed an increase of [^18^F]GTP1 standardized uptake value ratio (SUVR) of 3.5%−5.0% in the whole cortical gray (WCG) matter across study arms over 73 weeks in participants with prodromal and mild AD. Harrison et al. (2019) showed an annualized change in flortaucipir SUVR of 3.5%−7.0%, and Krishnadas et al. (2023) found an annual increase of 5%−7% in [^18^F]MK-6240 SUVR across several ROIs. To account for the differences in SUVR ranges between tracers, the percentage change in SUVR (%ΔSUVR) was estimated as the ratio of the published average annualized ΔSUVR and the average baseline values (Teng et al., 2022; Krishnadas et al., 2023).

Partial volume effects are a key factor that can impact longitudinal PET measurements. In the case of tau PET, if a region experiences no change in tau level but the region atrophies, a decrease of signal will be observed between baseline and follow-up. Atrophy accompanied by an increase in tau pathology could result in an increase, decrease, or no change in tau PET signal depending on the amount of tau and atrophy between baseline and follow-up. In addition, it has been shown that tau PET tracers exhibit various levels of off-target signal in white matter, extra-cortical regions, and some cortical regions that may change within the subject over time, resulting in variable levels of spill-in and spill-out of the measured signal across time points that can further complicate interpretation. Partial volume correction (PVC) is often applied to correct for various amounts of atrophy, signal blurring, and spillover from neighboring regions (Baker et al., 2017). Although PVC amplifies the signal, resulting in a broader dynamic range, it also amplifies the noise, making it necessary to test the impact of such methods on longitudinal measures of tau burden.

The primary goal of this work was to assess the impact of PVC methods on the quantification of longitudinal [^18^F]GTP1 tau PET using linear mixed-effects models (LMEM) to describe the SUVR temporal trajectories and estimate changes over time (Bernal-Rusie et al., 2012; Schwarz et al., 2021) by taking into consideration the dependency in the longitudinal measurements. LMEM allows the longitudinal within-subject variability and group separation of non-PVC and PVC data to be estimated. A second aim of this work was to evaluate the LMEM performance to estimate longitudinal change. We followed the approach used by Schwarz et al. (2019, 2021) to evaluate longitudinal flortaucipir and amyloid SUVR measurements.

## 2 Materials and methods

### 2.1 Participants

We analyzed longitudinal tau [^18^F]GTP1 PET imaging from both a natural history study (NHS) in cognitively unimpaired (CU) participants and those with AD (NCT02640092) and from a phase 2 clinical trial of semorinemab (an anti-tau antibody) in participants with prodromal to mild AD (NCT03289143). Primary data from these studies have been published elsewhere (Sanabria Bohórquez et al., 2019; Teng et al., 2019, 2021, 2022; Blennow et al., 2020; Barthélemy et al., 2022).

#### 2.1.1 Natural history study

We enrolled 63 participants between 50 and 85 years of age who were CU (*n* = 10) or had AD (*n* = 53) from 11 research centers. Baseline and longitudinal clinical assessments included the Mini-Mental State Examination (MMSE), Clinical Dementia Rating–Sum of Boxes (CDR), the 13-item version of the Alzheimer's Disease Assessment Scale-Cognitive Subscale, and the Repeatable Battery for the Assessment of Neuropsychological Status.

Participants with AD were further categorized into prodromal [mild cognitive impairment, MMSE 24–30; CDR global score 0.5; *n* = 24], mild (probable AD dementia; MMSE 22–30; CDR global score 0.5 or 1; *n* = 15), or moderate (probable AD dementia; MMSE 16– 21; CDR 0.5 or 1 or 2; *n* = 14) stages. Detailed inclusion criteria can be found in Teng et al. (2019). All participants in AD subgroups were required to have Aβ PET scans ([^18^F]florbetapir) adjudicated as positive via visual read and brain magnetic resonance imaging (MRI) scans that were consistent with AD and without significant non-AD neurological disease that might contribute to cognitive impairment. We aimed to include Aβ PET–positive and –negative participants in the CU group to capture a broad range of tau PET imaging in this group. All participants received [^18^F]GTP1 tau PET at baseline and had at least one follow-up scan.

#### 2.1.2 Semorinemab phase 2 in prodromal to mild AD (Tauriel)

Participants between 50 and 80 years of age who met the criteria for mild cognitive impairment or dementia due to AD were enrolled in the Tauriel study (Teng et al., 2022); 334 of the 422 enrolled study participants who received [^18^F]GTP1 tau PET at baseline and who had at least one follow-up scan are included in this work. All participants were required to have significant amyloid pathology indicated by Aβ PET scans ([^18^F]florbetaben, [^18^F]florbetapir, [^18^F]flutemetamol, or [^18^F]NAV4694) via visual reads or reduced cerebrospinal fluid (CSF) Aβ (1–42) levels. Cognitive assessments were performed at baseline and post-baseline at the week 49 and 73 visits. Baseline and longitudinal clinical assessments included the MMSE, the Clinical Dementia Rating–Sum of Boxes, the Alzheimer's disease assessment scale–cognitive subscale, the Repeatable Battery for the Assessment of Neuropsychological Status, and the Alzheimer's disease cooperative study–activities of daily living scale. Because no treatment differences were observed between the placebo arm or any of the semorinemab arms on clinical or [^18^F]GTP1 indices in the Tauriel study (Supplementary Figure S1), all treatment arms were combined for the analyses presented here.

### 2.2 Image acquisition and processing

MRI was performed at individual research centers on different 1.5T or 3T scanners for participant eligibility and imaging analyses. For the latter purposes, 3D Sagittal T1-weighted MPRAGE (magnetization-prepared rapid gradient-echo) or MPRAGE-like sequence adhering to the Alzheimer's Disease Neuroimaging Initiative specifications were collected with 1.0–1.2-mm slice thickness, 256-mm × 256-mm matrix, and a 240–270-mm field of view according to the manufacturer recommended acquisition parameters.

#### 2.2.1 Natural history study

[^18^F]GTP1 PET scans were performed at a central imaging center (Invicro, New Haven, CT). All participants were imaged with a Biograph 6 PET-CT, except for one participant with mild AD who was imaged using a Siemens HR+ PET. Images were reconstructed with an iterative reconstruction algorithm (OSEM 4 iterations, 16 subsets) and a *post hoc* 5-mm Gaussian filter for consistent image quality and data quantification across scanners. [^18^F]GTP1 tau PET imaging was performed at baseline and at the week 26, 52, and 78 post-baseline visits.

#### 2.2.2 Tauriel study

[^18^F]GTP1 tau PET imaging was performed in 79 imaging sites in North America, Europe, and Australia. PET technical site training and qualification were performed by a central vendor (Invicro) using Hoffman phantom data. PET images were corrected for attenuation, random coincidences, scatter, and isotope decay. Image reconstruction was completed using iterative/row-action maximum-likelihood algorithm methods with 4 iterations and 16 subsets (or as close as possible to 16) and a *post-hoc* Gaussian filter for obtaining consistent image quality and data quantification across scanners with a target resolution of 8 mm (Joshi et al., 2009). Participants underwent [^18^F]GTP1 tau PET at baseline and at weeks 49 and 73.

### 2.3 Neuroimaging methods

All MPRAGE scans were processed with FreeSurfer (version 6.0; http://surfer.nmr.mgh.harvard.edu/) to segment individual subjects' ROIs in native space using the Desikan–Killiany atlas. For each PET scan, the baseline and, when available, the closest-intime MRI were used with longitudinal FreeSurfer segmentation (Reuter et al., 2012). [^18^F]GTP1 SUVR was calculated using the inferior cerebellum gray matter as a reference region. To identify the inferior cerebellar gray matter, MPRAGE scans were non-linearly normalized to the MNI space using ANTs (version v2.4.3, https://github.com/ANTsX/ANTs). The calculated transformations were used to bring the SUIT cerebellum atlas (Diedrichsen, 2006.) from the MNI template space to the MPRAGE space. An ROI representing the inferior cerebellar cortex was generated from the SUIT cerebellum atlas and masked using the cerebellar mask obtained from FreeSurfer.

Anatomical target regions for the analysis were the composite ROIs included WCG and an AD-signature temporal ROI (TMP) comprising the entorhinal cortex; the amygdala; parahippocampal, fusiform, inferior and middle temporal cortices (Jack et al., 2017); and the hippocampus. In addition, the following individual cortical regions were considered: MT (mesial temporal regions: entorhinal cortex, amygdala, and hippocampus), modified TMP (mTMP, excluding MT), the rest of the temporal lobe, and the parietal, occipital, and frontal lobes.

Subjects were stratified according to their diagnostic group (CU, prodromal, mild, and moderate AD in the NHS, and prodromal and mild AD in the Tauriel study). We previously stratified participants with AD into low and high tau (Teng et al., 2021, 2022) and replicated observations of faster cognitive decline in subjects with higher tau burden (Pontecorvo et al., 2019; Betthauser et al., 2020). In this work, participants with AD in each study were stratified into three tau-level groups derived from the baseline TMP SUVR values quartiles to further interrogate the longitudinal change based on tau burden. Subjects in the first quartile were classified as a low-tau group and were expected to have the slowest tau accumulation overall. Subjects in the second and third quartiles were the mid-tau group, and subjects in the fourth quartile were the high-tau group.

### 2.4 PVC methods

We applied the 2-compartment PVC (Meltzer et al., 1990), which corrects primarily for differing levels of atrophy; 3-compartment PVC, which corrects for bleed-in from white matter and differing levels of atrophy (Müller-Gärtner et al., 1992); the van Cittert iterative deconvolution method (VC) with alpha = 1.0, 1.5, and 2.0 (Tohka and Reilhac, 2008), which does not rely on MRI segmentation and simply sharpens the image; and the region-based voxelwise method (RBV; Thomas et al., 2011) applied after geometric transfer matrix (GTM) PVC (Rousset et al., 1998; Baker et al., 2017), which corrects for bleed in and out of any regions defined.

### 2.5 Statistical methods

We used LMEM to describe the SUVR temporal trajectory (Bernal-Rusie et al., 2012; Schwarz et al., 2021) and estimate the annualized changes in tau burden over time. LMEMs account for the correlation among repeated measurements (i.e., within-subject and between-subject variability), with the assumption of linear individual trajectories. The predicted individual-specific response trajectories over time depend on the unknown variance among subjects and are shrunk toward the population-averaged mean response profile (Fitzmaurice et al., 2011).

We used logarithmic transformed SUVR (lnSUVR) values (with base *e*, i.e., natural logarithm) because the SUVR distributions tend to be right-skewed whereas lnSUVR tends to be more normally distributed. The LMEM applied to the NHS data included the fixed effects of *APOE4* status, age, sex, centiloids (CTLs), and time from baseline, as well as the interaction between time and group (diagnostic or tau-level groups) and the independent random effects terms for intercept and slope. The LMEM used in the fitting of the NHS data using Wilkinson–Rogers notation was lnSUVR ~ APOE4 + age + sex + CTL + time × Cohort + (1|subject) + (−1 + time|subject), where the lnSUVR was assumed to progress linearly over time, the progression rates were different across different diagnostic cohorts, and baseline *APOE4*, age, sex, and CTL were adjusted as confounding factors. The interaction between the diagnostic cohorts (CU, prodromal, mild, or moderate AD) and the AD tau-level groups (CU and AD subjects stratified in low tau, mid tau, and high tau) could not be performed because of the small sample size and the overlap of the two stratification approaches.

The LMEM applied to the Tauriel data was *lnSUVR* ~ APOE4 + age + sex + time × diagnostic cohort × tau level groups + (1|subject) + (−1 + time|subject), where the lnSUVR was assumed to progress linearly over time, the progression rates were different across different diagnostic cohorts and baseline tau-level groups, and baseline *APOE4*, age, and sex were adjusted as confounding factors. CTLs were not included in the Tauriel model because not all participants received an amyloid PET scan.

Another advantage of using lnSUVR is that the longitudinal within-subject variability or residual error for each PVC or non-PVC method can be calculated from the standard deviation (σ) of the residual or error term from the LMEM. The relationship between the σ and the variation coefficient of the original SUVR distribution is the percentage coefficient of variation ($(\%CV)=100\%\sqrt{e^{\sigma^{2}}-1}\approx 100\%\sigma \;$(Canchola et al., 2017), which is henceforth referred to as the residual error or within-subject variability. We followed Schwarz et al. (2021) approach to evaluate the PVC methods using two criteria: the within-subject variability as defined earlier and the longitudinal separability estimated from the *cohort* × *time* interaction *t*-scores term. Conceptually, this *t*-statistic term could be interpreted as a “longitudinal Cohen's *d*” effect size (ES) in that it is the ratio of group-wise differences over a measure of uncertainty and therefore provides an estimate of the separation in the SUVR slopes between the groups considered (CU vs. AD diagnostic groups or CU vs. AD tau burden–level groups). The relationship between both parameters was evaluated for no-PVC and PVC data, by which a PVC method that produces smaller within-subject variability (residual error) and greater longitudinal separability (*t*-scores) would be considered a better method for the data.

We compared the slopes of the fitted LMEM SUVR trajectories to the annualized change relative to baseline at each follow-up visit (calculated as the %ΔSUVR change divided by the elapsed time from baseline in years for each participant and visit). Because all participants received a tau PET at baseline and at least one follow-up visit, the LMEM allows the SUVR vs. time slopes for all subjects to be calculated. We calculated the longitudinal effect sizes as the mean SUVR percentage change divided by its standard deviation. The average and corresponding 95% confidence intervals of all measurements were estimated by applying bootstrap from 1,000 posterior simulations. All the analyses were performed in MATLAB (R2020a).

## 3 Results

### 3.1 Participants

Table 1 summarizes the demographic information of the subjects in the NHS and Tauriel studies. There was good agreement between Aβ PET visual reads and CTL quantification. In the NHS, 3 of 10 CU participants were Aβ PET–positive by visual reads. Quantification showed that 6 of the 10 CU participants and 52 of the 53 AD participants had CTL values above 26 (Amadoru et al., 2020). In the Tauriel study, 304 of the 314 subjects with a Aβ PET had CTL values above 26.

**Table 1:** Baseline demographic, cognitive, and neuroimaging characteristics of the cohorts in the NHS and Tauriel studies with baseline [^18^F]GTP1 imaging and at least one follow-up imaging visit.

	**Natural history study**				**Tauriel study**	
**Demographics**	**CU**	**Prodromal AD**	**Mild AD**	**Moderate AD**	**Prodromal AD**	**Mild AD**
Subjects, *N*	10	24	15	14	122	212
Female, *n* (%)	7 (70%)	16 (67%)	7 (47%)	4 (29%)	65 (53%)	116 (55%)
Age	67.2 (6.2)	69.5 (7.1)	73.1 (4.4)	70.4 (7.1)	70.2 (7.1)	69.1 (6.9)
MMSE	29.2 (0.8)	28.0 (1.5)	26.2 (2.8)	17.0 (2.8)	25.0 (2.4)	22.3 (2.4)
CDR-SB	0.1 (0.2)	1.6 (0.8)	3.4 (1.8)	6.3 (1.9)	2.6 (1.0)	4.4 (1.5)
ADAS-Cog13	9.3 (5.0)	14.8 (5.5)	21.4 (6.5)	40.2 (7.2)	24.6 (7.1)	30.2 (6.9)
RBANS	93.0 (10.8)	84.8 (12.1)	73.9 (15.1)	56.7 (9.9)	71.8 (10.8)	62.9 (9.7)
ADCS-ADL	–	–	–	–	71.9 (4.8)	66.6 (7.8)
APOE4 (carrier/noncarrier/unknown)	4/6/0	16/3/5	9/5/1	13/0/1	90/32	163/49
Amyloid centiloids^a^	37.4 (43.9)	77.7 (33.2)	77.1 (29.2)	87.4 (34.4)	90.7 (33.3)	89.5 (30.6)
TMP SUVR	1.28 (0.11)	1.37 (0.17)	1.63 (0.25)	1.91 (0.38)	1.49 (0.26)	1.59 (0.31)

Data are expressed as means (*SD*) unless otherwise specified.

AD, Alzheimer's disease; ADAS-Cog13, 13-item version of the Alzheimer's Disease Assessment Scale-Cognitive Subscale; ADCS-ADL, Alzheimer's Disease Cooperative Study Activities of Daily Living Scale; CDR-SB, Clinical Dementia Rating Sum of Boxes; CSF, cerebrospinal fluid; CU, cognitively unimpaired; MMSE, Mini-Mental State Examination; NHS, natural history study; PET, positron emission tomography; RBANS, Repeatable Battery for the Assessment of Neuropsychological Status Total Index; SUVR, standardized uptake value ratio; TMP, meta-temporal regions of interest. ^a^In the Tauriel study, 112 and 202 participants with prodromal and mild AD, respectively, received an amyloid PET scan; the rest of the subjects had amyloid positivity confirmed by measuring CSF Aβ(1-42) levels.

Table 2 shows the baseline regional SUVR in both studies. The participants with prodromal AD in the NHS were, on average, less cognitively impaired with a lower tau burden than the Tauriel participants, particularly in the MT region, with an SUVR of 1.37 (95% CI [1.31, 1.46]) and 1.50 (95% CI [1.45, 1.56]), respectively. Of the 24 prodromal participants in the NHS, 13 would have met the Tauriel study inclusion criteria, which required significant impairment in episodic memory performance.

**Table 2:** Regional baseline SUVR in the NHS and Tauriel studies.

**Region, average (95% CI)**	**NHS**				**Tauriel**	
	**CU**	**Prodromal**	**Mild**	**Moderate**	**Prodromal**	**Mild**
	***n*** = **10**	***n*** = **24**	***n*** = **15**	***n*** = **14**	***n*** = **122**	***n*** = **212**
MT	1.30 [1.19, 1.38]	1.36 [1.29, 1.43]	1.47 [1.38, 1.58]	1.63 [1.49, 1.83]	1.45 [1.42, 1.49]	1.49 [1.46, 1.51]
mTMP	1.28 [1.21, 1.35]	1.37 [1.31, 1.46]	1.67 [1.54, 1.82]	1.98 [1.74, 2.19]	1.50 [1.45, 1.56]	1.62 [1.57, 1.67]
Rest of temporal	1.16 [1.11, 1.27]	1.18 [1.13, 1.23]	1.32 [1.24, 1.42]	1.54 [1.33, 1.71]	1.23 [1.20, 1.27]	1.30 [1.27, 1.33]
Parietal	1.16 [1.10, 1.23]	1.19 [1.14, 1.26]	1.35 [1.27, 1.46]	1.62 [1.38, 2.05]	1.27 [1.23, 1.33]	1.36 [1.31, 1.41]
Occipital	1.24 [1.19, 1.31]	1.26 [1.21, 1.33]	1.39 [1.30, 1.49]	1.60 [1.40, 1.91]	1.31 [1.27, 1.36]	1.38 [1.34, 1.42]
Frontal	1.09 [1.03, 1.16]	1.08 [1.05, 1.12]	1.17 [1.12, 1.24]	1.33 [1.16, 1.65]	1.13 [1.10, 1.16]	1.16 [1.13, 1.19]
TMP	1.28 [1.20, 1.35]	1.37 [1.31, 1.44]	1.63 [1.52, 1.77]	1.91 [1.72, 2.10]	1.49 [1.44, 1.53]	1.59 [1.55, 1.64]
WCG	1.16 [1.11, 1.23]	1.18 [1.14, 1.23]	1.32 [1.26, 1.41]	1.52 [1.35, 1.80]	1.24 [1.21, 1.28]	1.30 [1.27, 1.34]

CU, cognitively unimpaired; MT, mesial temporal entorhinal cortex, hippocampus, and amygdala; mTMP, TMP excluding MT; NHS, natural history study; SUVR, standardized uptake value ratio; TMP, temporal meta-regions of interest; WCG, whole cortical gray.

Average and 95% confidence intervals were estimated applying bootstrap.

In the NHS, the average (± standard deviation) time interval between baseline and the scheduled scans at weeks 26, 52, and 78 was 26.5 ± 2.5 (*n* = 63), 53.9 ± 3.23 (*n* = 56), and 78.7 ± 3.6 weeks (*n* = 51), respectively. In the Tauriel study, the average (± standard deviation) time interval between baseline and the scheduled scans at weeks 49 and 73 was 50.6 ± 3.35 (*n* = 314) and 75.3 ± 3.8 weeks (*n* = 288), respectively.

### 3.2 Tau levels

**Figure 1:**
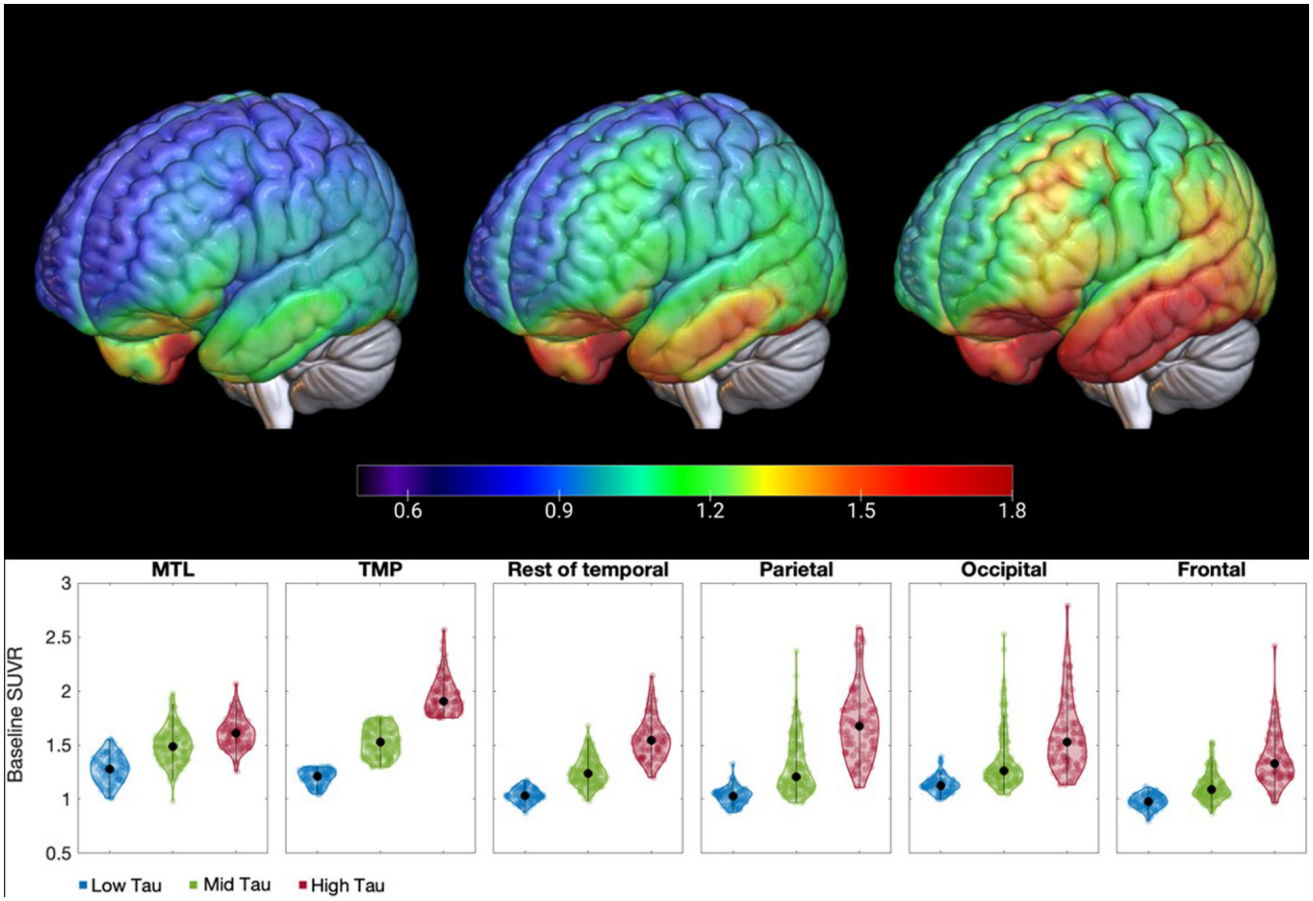
Surface maps and violin plots of [^18^F]GTP1 SUVR displaying the distribution of tau burden by tau level at baseline in the Tauriel study in prodromal and mild AD participants: low tau (TMP SUVR ≤1.30), mid tau (1.30 < TMP SUVR ≤ 1.75) and high tau (TMP SUVR >1.75). The pattern of the uptake from low to high tau burden in the temporal meta-regions of interest is maintained across the cortex. AD, Alzheimer's disease; MT, mesial temporal regions; SUVR, standardized uptake value ratio; TMP, temporal meta-regions of interest.

Table 3 shows the baseline TMP-defined cut points between tau levels for both studies based on the quartiles. The Tauriel SUVR cut points between low and mid tau burden and between mid and high tau for the Tauriel data were 3% and 1.5% lower than the corresponding cut points for the NHS data. Figure 1 shows the surface maps of average [^18^F]GTP1 SUVR by tau level at baseline in the Tauriel study in participants with prodromal and mild AD.

**Table 3:** AD participant SUVR cut points for the first and third quartiles in the baseline meta-temporal region of interest.

**Study**	**PVC method**	**SUVR 1st and 3rd quartile cut points**	
		**25%**	**75%**
NHS AD	Non-PVC	1.34	1.78
	PVC-2 compartments	1.71	2.47
	PVC-3 compartments	1.92	2.87
	PVC-VC10	1.72	2.79
	PVC-VC15	1.36	1.92
	PVC-VC20	1.36	1.93
	PVC-RBV	1.36	1.95
Tauriel	Non-PVC	1.30	1.75

AD, Alzheimer's disease; NHS, natural history study; PVC, partial volume correction; RBV, region-based voxelwise; SUVR, standardized uptake value ratio; VC, van Cittert iterative deconvolution.

### 3.3 Evaluation of PVC methods

**Figure 2:**
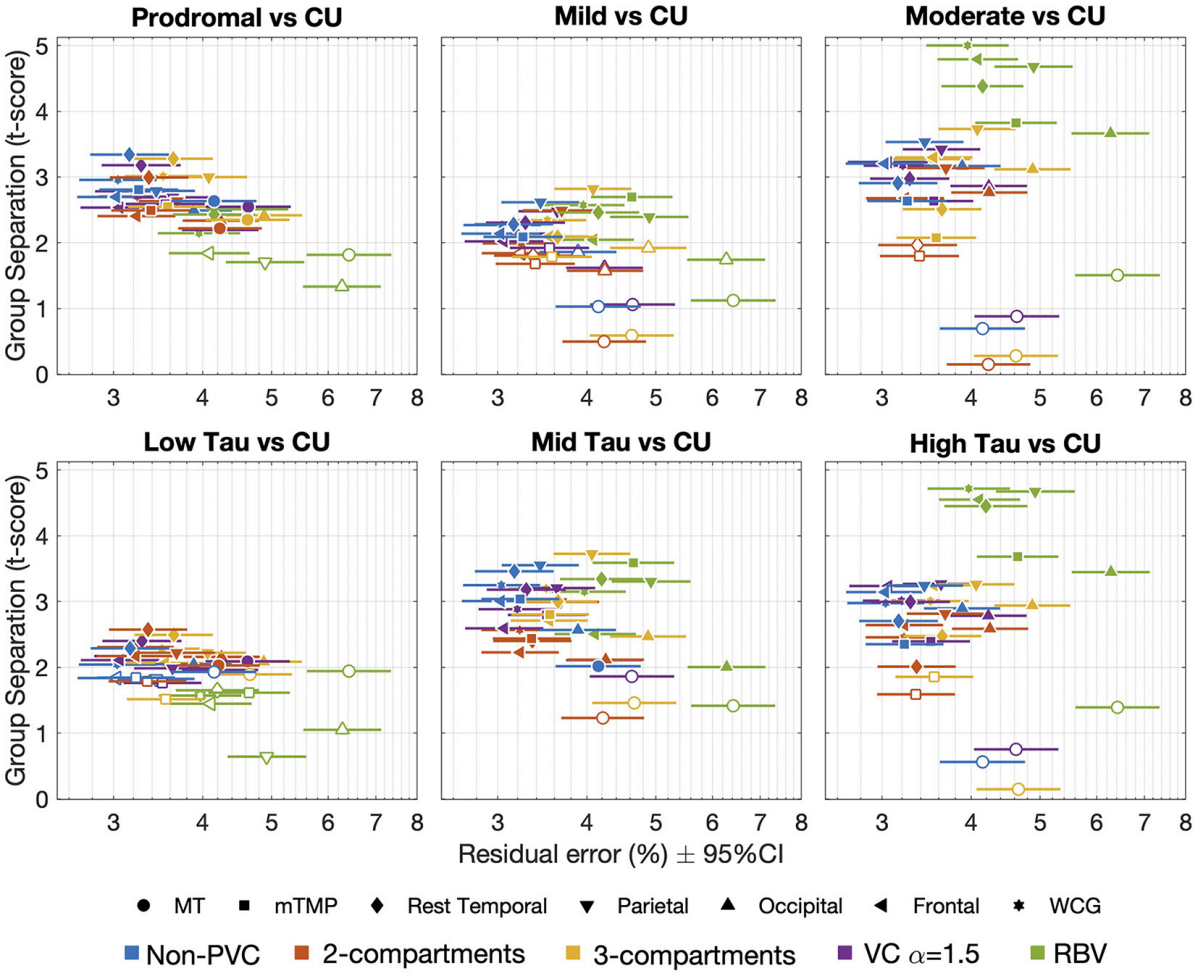
Relationship between the measure of group separation (*cohort* × *time* interaction *t*-score) and the within-subject variability or residual error. Each point in the plot shows the *t*-score and residual error in a given region (symbols) for a given PVC method (or non-PVC). Filled symbols indicate significant differences between AD groups (diagnostic groups on top and tau-level groups in the bottom row) and cognitively unimpaired participants (*t*-scores >1.96, *p* < 0.05). CU, cognitively unimpaired; MT, mesial temporal regions; mTMP, PVC, partial volume correction; RBV, region-based voxelwise; TMP excluding MT; VC, van Cittert iterative deconvolution; WCG, whole cortical gray.

Figure 2 displays the relationship between the measure of group separation (t-score) and the within-subject variability (residual error; Supplementary Tables S1, S2). The region with the larger residual errors and smaller t-scores was the MT with all methods. The largest within-subject variability was observed with PVC-RBV across regions, from 4.0% (95% CI [3.5, 4.5]) residual error in the WCG to 6.5% (95% CI [5.6, 7.4]) in the MT. The lowest variability was observed for non-PVC data from 3.0% (95% CI [2.7, 3.4]) residual error in the WCG to 4.1% (95% CI [3.6, 4.8]) in the MT. The variability of the other PVC methods was between 3.2% and 4.6%. The largest t-scores for the moderate AD vs. CU or high-tau AD vs. CU comparisons were observed for PVC-RBV. Data without PVC displayed superior or similar t-scores for the separation between prodromal or mild AD and low- or mid-tau AD and CU and mild AD vs. CU. In general, lower within-subject variability and significant group separation t-scores were observed without PVC. The results of the VC-PVC were very similar to the three alpha values considered (1.0, 1.5, and 2.0); therefore, henceforth, we include only results from the VC-PVC (alpha = 1.5).

The largest *t*-score for the low tau or prodromal vs. CU was observed in the temporal cortex subregion, excluding TMP (2.29 and 3.34, respectively). The region with the larger *t*-scores for the other comparisons was the parietal cortex with other comparisons: mid and high tau vs. CU scores of 3.55 and 3.25, respectively, and mild or moderate AD vs. CU scores of 2.61 and 3.53, respectively.

A higher tau burden has been associated with more atrophy (La Joie et al., 2020; Das et al., 2021; also see Supplementary Figure S2) and PVC is expected to help correct for the impact of atrophy on SUVR. VC-PVC and non-PVC SUVR values were similar whereas higher SUVRs were obtained from the 2-compartment, 3-compartment, and RBV images (Supplementary Figure S3). In the CU, low tau, and prodromal AD participants, the annualized %ΔSUVR was similar for the non-PVC and PVC data with some exceptions, with a lower change observed in participants with AD when using PVC-RBV (Supplementary Figure S4). Although the annualized PVC-RBV %ΔSUVR was significantly higher than any other method in the high-tau and moderate AD groups, the differences between the 2-compartment or 3-compartment and PVC-RBV suggest RBV may be overcorrecting regions with higher tau burden. None of the PVC models displayed a consistently larger longitudinal ES across regions and diagnostic or tau-level groups. Overall, the annualized non-PVC %ΔSUVR displayed a longitudinal ES ± 95% CI similar to PVC methods (Supplementary Figure S5). In the parietal region, the 3-compartment PVC method displayed a larger longitudinal ES but with a wider 95% CI.

Further analyses were performed in order to better understand the increase in annualized %ΔSUVR seen in the high-tau group using the PVC-RBV method. White matter and CSF are the two regions with the largest partial volume effects impact on the cortex. These are both accounted for in the 3-compartment method (white matter is the mean of eroded non-PVC white matter, CSF = 0) and PVC-RBV (CSF is non-zero). We therefore looked at the annualized %ΔSUVR in white matter (eroded non-PVC and PVC-RBV) and CSF (non-PVC and PVC-RBV, defined by SPM12 segmentation) across different tau levels (low, medium, high). Within each tau level, there was no significant difference between non-PVC and PVC-RBV annualized %ΔSUVR in white matter. However, in CSF, only the high-tau group showed a negative annualized %ΔSUVR change, and this was significantly more negative than in RBV. In PVC, as the CSF signal decreases, the amplification of the cortical signal increases. Therefore, the decrease in CSF annualized %ΔSUVR in PVC-RBV (vs. stable CSF annualized %ΔSUVR in 3-compartment PVC) would result in an increase in annualized %ΔSUVR in the cortex.

All the images in the NHS were acquired at a single site with all subjects but one undergoing imaging in the same scanner, which provides a robust data set for evaluating PVC methods. Our results show that PVC methods do not offer a clear advantage or improvement over no-PVC images for the quantification of longitudinal [^18^F]GTP1 SUVR change. All subsequent analyses presented here were performed without applying PVC to the PET data.

### 3.4 Longitudinal [^18^F]GTP1 SUVR change

**Figure 3:**
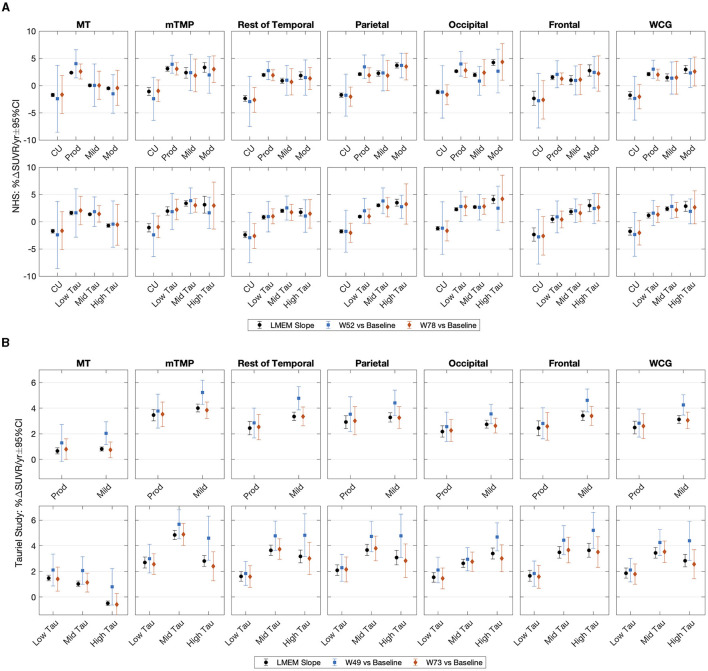
Average annualized change in SUVR and the corresponding 95% confidence intervals in the **(A)** NHS participants and **(B)** the Tauriel study estimated from the LMEM slope analysis and at the follow up visits (NHS: weeks 52 and 78; Tauriel: weeks 49 and 73) relative to baseline. Subjects are grouped by diagnostic cohort or tau level. Average and confidence intervals were estimated applying bootstrap. %ΔSUVR, standardized uptake value ratio change; CU, cognitively unimpaired; LMEM, linear mixed effects models; MT, mesial temporal regions; mTMP, NHS, natural history study; PVC, partial volume correction; RBV, region-based voxelwise; SUVR, standardized uptake value ratio; TMP excluding MT; VC, van Cittert iterative deconvolution; W, week; WCG, whole cortical gray.

In the NHS, we found good numerical agreement between the average annualized %ΔSUVR estimated when applying LMEM to describe the SUVR trajectories and the annualized average of percentage change at the visits during weeks 52 and 78 (Figure 3). The SUVR change from the LMEM slope analysis had narrower 95% CIs, resulting in larger regional longitudinal ES than for the visit averages (Supplementary Figure S6). For example, when grouping subjects by tau level, the WCG LMEM %ΔSUVR/year ES was between 1.30 (95% CI [0.49, 2.13]) and 2.12 (95% CI [1.35, 2.68]) compared to an ES range between 0.45 (95% CI [−0.25, 1.05]) and 0.64 (95% CI [−0.19, 2.23]) as calculated using annualized week 78 SUVR changes. The regional averages of LMEM SUVR slopes and the longitudinal ES are shown in Table 4 (Supplementary Table S3 for the per-visit averages).

**Table 4:** Natural history study: average annualized %ΔSUVR (without partial volume correction) and corresponding longitudinal effect size.

**Region**	**NHS LMEM slope analysis %**Δ**SUVR/year [95% CI]**							
	**CU**	**Prodromal AD**	**Mild AD**	**Moderate AD**	**CU**	**Low tau**	**Mid tau**	**High tau**
	***n*** = **10**	***n*** = **24**	***n*** = **15**	***n*** = **14**	***n*** = **10**	***n*** = **13**	***n*** = **30**	***n*** = **10**
MT	−1.70 [−2.02, −1.38]	2.37 [2.25, 2.50]	0.04 [−0.20, 0.27]	−0.52 [−0.75, −0.32]	−1.75 [−2.11, −1.37]	1.61 [1.25, 1.91]	1.37 [1.21, 1.52]	−0.72 [−1.02, −0.40]
mTMP	−1.07 [−1.81, −0.41]	3.10 [2.64, 3.61]	2.35 [1.36, 3.48]	3.33 [2.43, 4.01]	−1.11 [−1.89, −0.42]	1.94 [1.17, 2.71]	3.39 [2.85, 4.00]	3.11 [1.59, 4.29]
Rest of temporal	−2.37 [−2.90, −1.85]	1.96 [1.71, 2.26]	0.88 [0.39, 1.45]	1.83 [1.15, 2.22]	−2.41 [−2.93, −1.93]	0.83 [0.45, 1.26]	1.99 [1.69, 2.24]	1.72 [1.04, 2.24]
Parietal	−1.75 [−2.13, −1.44]	2.09 [1.85, 2.44]	2.24 [1.75, 2.75]	3.72 [3.23, 4.03]	−1.77 [−2.10, −1.49]	0.94 [0.72, 1.21]	3.00 [2.73, 3.29]	3.47 [2.84, 3.88]
Occipital	−1.19 [−1.53, −0.80]	2.64 [2.44, 2.89]	1.95 [1.60, 2.35]	4.24 [3.70, 4.75]	−1.24 [−1.59, −0.83]	2.29 [1.98, 2.70]	2.68 [2.45, 2.93]	4.08 [3.30, 4.80]
Frontal	−2.35 [−3.65, −1.45]	1.50 [1.10, 2.14]	1.00 [0.14, 1.95]	2.76 [1.72, 3.68]	−2.35 [−3.58, −1.50]	0.47 [−0.20, 1.07]	1.82 [1.30, 2.37]	2.93 [1.84, 4.13]
TMP	−1.22 [−1.93, −0.50]	2.96 [2.56, 3.39]	2.00 [1.10, 3.15]	2.63 [1.73, 3.28]	−1.27 [−2.03, −0.55]	1.89 [1.06, 2.70]	3.04 [2.51, 3.65]	2.45 [1.21, 3.78]
WCG	−1.77 [−2.46, −1.20]	2.09 [1.76, 2.48]	1.48 [0.82, 2.25]	2.96 [2.23, 3.53]	−1.80 [−2.49, −1.29]	1.14 [0.68, 1.62]	2.39 [2.02, 2.80]	2.83 [1.90, 3.70]
**Region**	**NHS LMEM slope analysis %**Δ**SUVR/year effect size [95% CI]**							
	**CU**	**Prodromal AD**	**Mild AD**	**Moderate AD**	**CU**	**Low tau**	**Mid tau**	**High tau**
	***n*** = **10**	***n*** = **24**	***n*** = **15**	***n*** = **14**	***n*** = **10**	***n*** = **13**	***n*** = **30**	***n*** = **10**
MT	−3.46 [−5.07, −2.28]	7.43 [5.61, 9.05]	0.08 [−0.50, 0.67]	−1.26 [−1.84, −0.42]	−3.11 [−4.61, −2.11]	2.73 [1.80, 4.00]	3.17 [2.36, 4.00]	−1.43 [−2.36, −0.38]
mTMP	−0.95 [−1.71, −0.33]	2.74 [2.10, 3.42]	1.11 [0.61, 1.66]	2.23 [1.26, 3.57]	−0.95 [−1.68, −0.39]	1.32 [0.66, 2.03]	2.14 [1.52, 2.74]	1.44 [0.66, 2.68]
Rest of temporal	−2.78 [−4.20, −1.35]	2.94 [2.06, 3.93]	0.85 [0.31, 1.38]	1.86 [0.94, 3.10]	−3.02 [−4.51, −1.51]	1.14 [0.31, 1.93]	2.53 [1.80, 3.26]	1.82 [1.09, 3.13]
Parietal	−3.05 [−4.20, −1.64]	2.83 [2.09, 3.77]	2.12 [1.04, 3.17]	5.02 [3.23, 8.49]	−3.55 [−4.89, −2.01]	2.13 [1.30, 2.94]	3.73 [2.46, 4.89]	4.55 [3.17, 8.08]
Occipital	−2.09 [−3.40, −1.17]	4.56 [3.44, 5.84]	2.47 [1.69, 3.18]	4.17 [2.53, 6.00]	−2.04 [−3.48, −1.10]	3.34 [2.16, 4.75]	3.84 [2.86, 4.73]	3.24 [1.76, 4.98]
Frontal	−1.34 [−2.22, −0.44]	1.16 (0.62–1.64)	0.55 [0.00, 1.07]	1.49 [0.64, 2.36]	−1.41 [−2.33, −0.50]	0.42 [−0.32, 1.13]	1.17 [0.69, 1.60]	1.58 [0.98, 2.16]
TMP	−1.04 [−1.89, −0.35]	2.84 [2.18, 3.57]	1.02 [0.52, 1.56]	1.86 [0.96, 3.19]	−1.05 [−1.81, −0.40]	1.27 [0.60, 2.07]	2.04 [1.42, 2.62]	1.23 [0.47, 2.32]
WCG	−1.79 [−2.60, −0.71]	2.41 [1.85, 3.11]	1.07 [0.43, 1.56]	2.45 [1.39, 4.06]	−1.85 [−2.66, −0.76]	1.31 [0.51, 2.14]	2.15 [1.37, 2.72]	1.97 [1.21, 3.27]

%ΔSUVR, standardized uptake value ratio change; AD, Alzheimer's disease; CU: cognitively unimpaired; LMEM, linear mixed-effects models; MT, mesial temporal entorhinal cortex, hippocampus, and amygdala; mTMP, TMP excluding MT; NHS, natural history study; SUVR, standardized uptake value ratio; TMP, temporal meta-regions of interest; WCG, whole cortical gray. Average and 95% confidence intervals were estimated by applying bootstrap.

Taking into consideration that the [^18^F]GTP1 tau PET in Tauriel was a multisite imaging study, the variability of PVC methods can be expected to be much larger than what we observed in the NHS because of the wider range of scanners with variable spatial resolution. Therefore, the data presented here are reconstructed using the harmonized approach for multisite imaging without any partial volume correction.

Figure 3 shows the average annualized %ΔSUVR estimated using LMEM to describe the SUVR trajectories and the annualized average of change at visit weeks 49 and 73 in the Tauriel study. Regional averages of LMEM SUVR slopes and the longitudinal ES are shown in Table 5 (per-visit calculations in Supplementary Table S4 and Supplementary Figures S7, S8). Consistent with the NHS observations, the LMEM slope analysis longitudinal CIs were smaller (and resulting ES was larger) than for the visit averages (Figure 3). For example, when stratifying subjects by tau level, the WCG LMEM %ΔSUVR/year ES was between 0.99 (95% CI [0.74, 1.22]) and 1.35 (95% CI [0.84, 1.59]) compared to 0.50 (95% CI [0.22, 0.71]) and 0.68 (95% CI [0.42, 0.86]) at week 73.

**Table 5:** Tauriel study: average annualized %ΔSUVR estimated from the LMEM SUVR trajectories and the corresponding effect size calculated as the ratio of the mean and the standard deviation.

**Region**	**Tauriel LMEM slope analysis %**Δ**SUVR/year [95% CI]**				
	**Prodromal AD**	**Mild AD**	**Low tau**	**Mid tau**	**High tau**
	***n*** = **122**	***n*** = **212**	***n*** = **82**	***n*** = **169**	***n*** = **83**
MT	0.66 [0.37, 0.87]	0.81 [0.62, 0.97]	1.48 [1.27, 1.69]	1.04 [0.81, 1.19]	−0.49 [−0.69, −0.29]
mTMP	3.46 [2.91, 3.93]	4.04 [3.74, 4.42]	2.71 [2.35, 3.27]	4.90 [4.49, 5.27]	2.82 [2.35, 3.30]
Rest of temporal	2.46 [1.92, 2.88]	3.38 [3.03, 3.73]	1.61 [1.18, 2.01]	3.70 [3.25, 4.09]	3.21 [2.68, 3.82]
Parietal	2.93 [2.41, 3.42]	3.31 [2.96, 3.66]	2.11 [1.71, 2.61]	3.73 [3.27, 4.17]	3.11 [2.55, 3.68]
Occipital	2.20 [1.82, 2.61]	2.77 [2.48, 3.06]	1.54 [1.15, 2.02]	2.64 [2.27, 2.93]	3.42 [2.98, 3.86]
Frontal	2.47 [1.96, 3.06]	3.46 [3.10, 3.84]	1.66 [1.24, 2.22]	3.52 [3.04, 3.92]	3.68 [3.04, 4.25]
TMP	2.98 [2.50, 3.38]	3.47 [3.16, 3.81]	2.50 [2.15, 3.02]	4.22 [3.75, 4.56]	2.25 [1.77, 2.76]
WCG	2.51 [2.01, 2.95]	3.15 [2.86, 3.50]	1.87 [1.46, 2.32]	3.48 [3.01, 3.84]	2.87 [2.39, 3.37]
**Region**	**Tauriel LMEM slope analysis %**Δ**SUVR/year effect size** ±**95% CI**				
	**Prodromal AD**	**Mild AD**	**Low tau**	**Mid tau**	**High tau**
	***n*** = **122**	***n*** = **212**	***n*** = **82**	***n*** = **169**	***n*** = **83**
MT	0.52 [0.14, 0.73]	0.64 [0.49, 0.78]	1.55 [1.25, 1.82]	0.91 [0.39, 1.15]	−0.56 [−0.81, −0.30]
mTMP	1.37 [0.77, 1.62]	1.68 [1.47, 1.87]	1.37 [1.06, 1.59]	2.06 [1.25, 2.47]	1.30 [1.02, 1.55]
Rest of temporal	1.00 [0.52, 1.29]	1.32 [1.12, 1.46]	0.85 [0.60, 1.03]	1.45 [0.91, 1.73]	1.24 [0.93, 1.48]
Parietal	1.04 [0.61, 1.28]	1.24 [1.04, 1.40]	1.04 [0.73, 1.24]	1.28 [0.76, 1.50]	1.21 [0.86, 1.50]
Occipital	1.04 [0.57, 1.28]	1.34 [1.15, 1.51]	0.85 [0.58, 1.07]	1.28 [0.78, 1.51]	1.70 [1.39, 1.98]
Frontal	0.86 [0.48, 1.06]	1.22 [1.06, 1.39]	0.76 [0.52, 0.95]	1.15 [0.74, 1.35]	1.40 [1.07, 1.68]
TMP	1.24 [0.71, 1.53]	1.51 [1.33, 1.72]	1.29 [0.95, 1.48]	1.85 [1.19, 2.21]	1.08 [0.82, 1.31]
WCG	1.03 [0.55, 1.27]	1.32 [1.13, 1.48]	0.99 [0.74, 1.22]	1.35 [0.84, 1.59]	1.25 [0.95, 1.52]

%ΔSUVR, standardized uptake value ratio change; AD, Alzheimer's disease; LMEM, linear mixed effects models; MT, mesial temporal entorhinal cortex, hippocampus, and amygdala; mTMP, TMP excluding MT; SUVR, standardized uptake value ratio; TMP, temporal meta-regions of interest; WCG, whole cortical gray. Average and 95% confidence intervals were estimated applying bootstrap.

The annualized week 73 SUVR changes and the LMEM SUVR slopes were similar across target regions, but both measurements were numerically lower than the annualized week 49 SUVR change, particularly in the mild AD group or the mid- to high-tau groups. The difference could be explained, at least in part, by SUVR increases at week 49 followed by decreases at week 73. The percentage of participants displaying a larger SUVR increase at week 49 was about 36% in both placebo and semorinemab arms; 35% and 37% in prodromal and mild AD, respectively; and 28%, 37%, and 43% in the low-, mid-, and high-tau groups, respectively. A similar percentage of NHS participants displayed increases followed by subsequent decreases during the 78 weeks of observations (SUVR plots as a function of age are shown in Supplementary Figures S7, S9).

**Figure 4:**
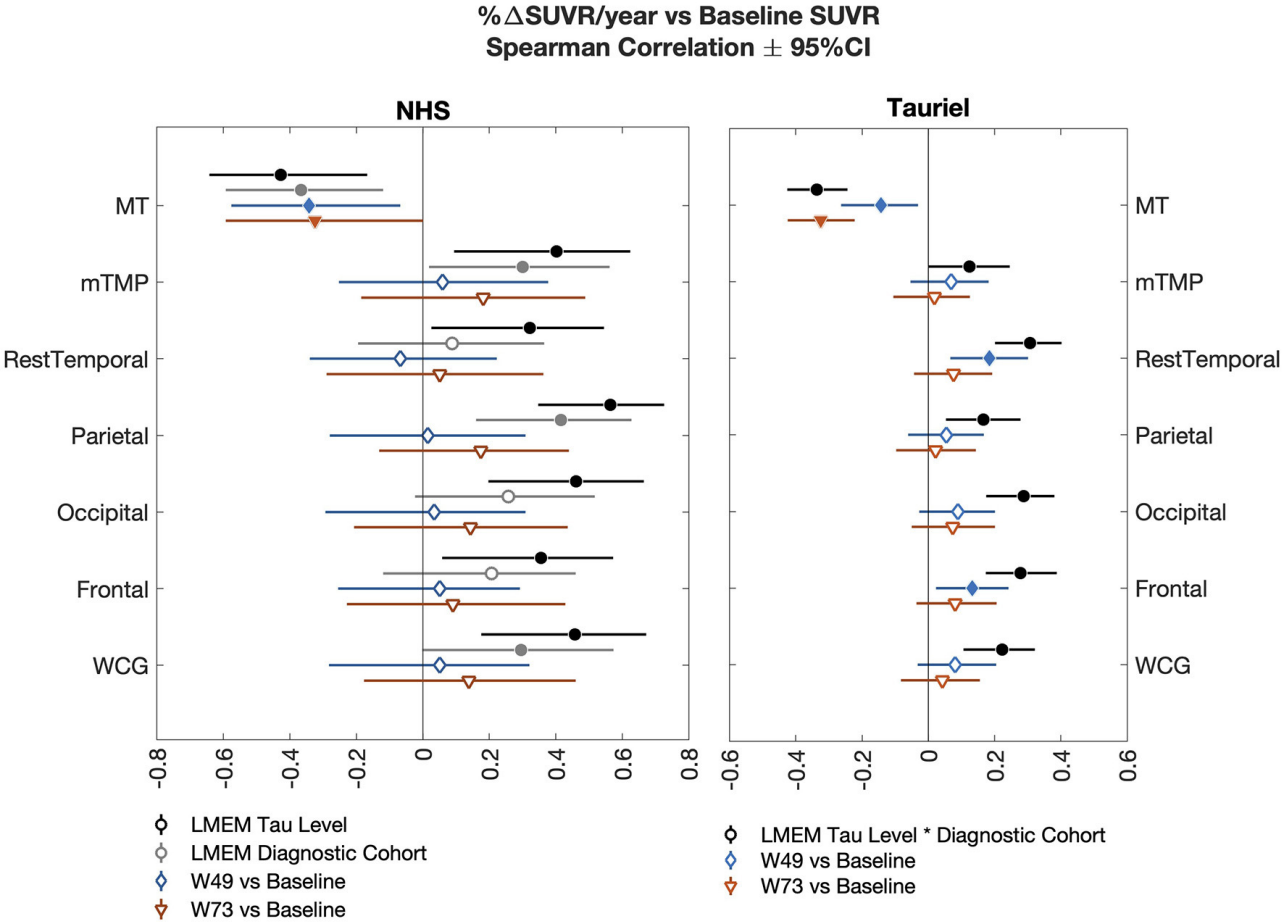
Forest plots illustrate the Spearman correlations (±95% CI) between annualized SUVR change and baseline SUVR in the NHS (AD participants only; **left**) and the Tauriel study **(right)** across different regions of interest. In the NHS, the two LMEM slope analysis including *tau level* × *time* or *diagnostic* × *time* interactions are shown. The Tauriel LMEM included the *Diagnostic* × *tau level* × *time* interaction. AD, Alzheimer's disease; LMEM, linear mixed-effects models; MT, mesial temporal regions; mTMP, TMP excluding MT; NHS, natural history study; SUVR, standardized uptake value ratio; W, week; WCG, whole cortical gray.

**Figure 5:**
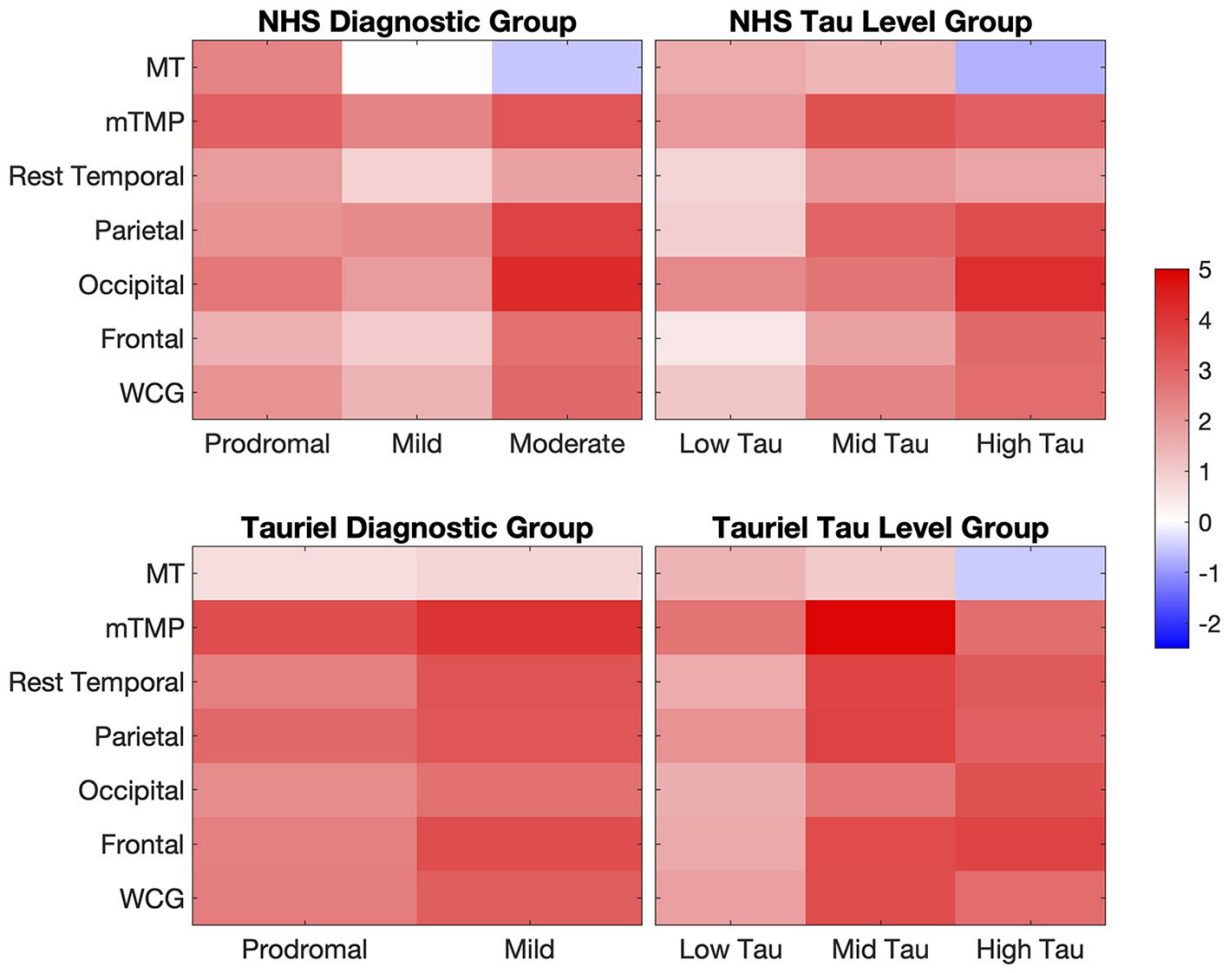
Heat maps of the annualized %ΔSUVR estimated from the LMEM slope analysis in AD participants in the NHS and Tauriel study. %ΔSUVR, standardized uptake value ratio change; AD, Alzheimer's disease; LMEM, linear mixed-effects models; MT, mesial temporal regions; mTMP, TMP excluding MT; NHS, natural history study; WCG, whole cortical gray.

Overall, the longitudinal tau accumulation varies as a function of the baseline tau burden. Figure 4 shows the forest plots with the Spearman correlations between baseline [^18^F]GTP1 SUVR and %ΔSUVR/year in AD participants across different ROIs. In the NHS, a stronger correlation between baseline and longitudinal change was observed when including a *tau level* × *time* interaction term than the *diagnostic* × *time* interaction term in the LMEM. In both studies, although we observed a negative correlation between baseline SUVR and %ΔSUVR/year in MT (Figure 4), baseline SUVR was prognostic of its own change from baseline in other regions. On average, small changes or decreases in SUVR were observed in the MT in the mild-to-moderate AD participants and the high-tau group without PVC and after 2-compartment, 3-compartment, and VC-PVC (see Supplementary Figure S4). Therefore, the loss of signal implied by the negative correlation may not entirely reflect atrophy. The larger tau burden increases in the MT were observed in the NHS prodromal AD and low- to mid-tau participants. On average, a negative SUVR change was observed in the NHS CU group, but the small number of CU participants prevents a clear interpretation of these findings. The two studies' regional changes are shown in the heat maps of annualized %ΔSUVR in Figure 5.

## 4 Discussion

PVC is commonly applied to amyloid and tau PET data in aging and dementia research because of varying atrophy levels. Although PVC increases the signal, it also increases the noise. In postprocessing PVC methods, there are often some violations of the assumptions that underlie these approaches. For both 2-compartment and 3-compartment PVC, we assume that the signal outside of the cortex is 0. For 3-compartment PVC, we are assuming that the white matter is homogeneous and that the estimation of white matter value (calculated from an eroded white matter region) is equal to that of white matter free of partial volume effects. The version of GTM we implemented does not assume the signal outside the cortex is zero; instead, it includes extra-cortical ROIs, CSF, skull, and meninges in the model (Baker et al., 2017). However, the use of GTM still assumes that regions are homogeneous, and if too many regions are included in the model, it can be ill conditioned. RBV can make small adjustments to homogeneous regions, but the effect of RBV on the overall mean of a region is small. In addition, all these PVC methods rely on segmentation of the MRI and accurate co-registration of the PET SUVR to the MRI. VC-PVC is the only method we tested that does not rely on the MRI; however, VC-PVC and non-PVC displayed similar performance.

The apparent overcorrection of RBV on regions with high tau in comparison to 2-compartment or 3-compartment PVC appears to be due to a decrease in the CSF signal over time (seen in both non-PVC and PVC-RBV) for the high-tau group (no decrease seen in the low- or medium-tau group). The decrease in the CSF signal results in a greater amplification of the cortical signal, which is not seen in 2-compartment or 3-compartment PVC since both methods assume the CSF signal is always 0. With any data analysis method, any step added could add noise. Our attempt to carefully correct for extra cortical signal bleed in (GTM/RBV) could have resulted in the addition of more noise than signal. It is advisable to use the simplest data analysis method needed. In this case, the PVC methods do not result in value gained in the analysis, and in the case of RBV could result in value loss. This study was done on a single scanner wherein the effective resolution remained the same and the reconstruction methods were easily tracked, and there was no value added with PVC. This leads us to conclude that complicating multisite analyses with PVC would not improve the signal and that proceeding without partial volume correction when analyzing [^18^F]GTP1 longitudinal data is advisable. Furthermore, the 3%−4% within-subject longitudinal variability of [^18^F]GTP1 SUVR measurements was similar to the variability observed in repeated imaging in CU and AD participants (Sanabria Bohórquez et al., 2019), supporting the use of non-PVC images.

Comparable to our results (Supplementary Figure S4), Jack et al. (2020) found that in cognitive impaired amyloid–positive subjects (*n* = 134, median visit interval of 1.3 years), flortaucipir SUVR changes calculated on non-PVC or 2-compartment PVC data using LMEM were similar. In contrast, in a similar population (*n* = 30, median visit interval of 1.1 years), Jack et al. (2018) found larger and more variable SUVR changes after applying 2-compartment PVC and calculating change as the difference in flortaucipir SUVR between two visits divided by time between scans. In a detailed analysis of target and references regions and PVC methods, Schwarz et al. (2021) analyzed [^18^F]flortaucipir PET scans on 97 subjects (46 unimpaired, 51 impaired) with an average of 2.5 years between baseline and the third tau PET scan. They found that the implementation of GTM, where extra-cortical hot spots are defined (the same as used in this article), increased the group separation the most (in comparison to no PVC, 2-compartment, 3-compartment, and GTM with no extra-cortical hot spots defined), but it also harmed the repeatability. However, repeatability was measured as the within-participant variation, and it is not clear if minimizing this reflects a more accurate quantification of the underlying PET SUVR values before partial volume effects. PVC methods need to be evaluated and validated for multisite imaging settings for any other PET ligand because the differences in the distribution of off-target signals may play a role in the PVC performance.

We successfully adapted the methodology proposed by Schwarz et al. (2021) to evaluate PVC approaches to longitudinal [^18^F]GTP1 tau PET in a modest sample size. Furthermore, our results show that the modeling of longitudinal data using LMEM is a robust approach that straightforwardly allows data from participants with missing time points or variable time between observations to be included.

Although annualized SUVR changes from the LMEM slope analysis were numerically comparable to per-visit estimates, the variability was lower, with larger longitudinal effect sizes and an improved relationship between baseline SUVR and annualized SUVR change. The lower increases relative to baseline in tau burden at week 73 than at week 49 in 36% of participants in the Tauriel study was not related to the treatment arm (placebo vs. semorinemab; Supplementary Figure S10), and it is very unlikely the differences reflect changes in regional or global cerebral blood flow (Sanabria Bohórquez et al., 2020). Tau changes over a 6-month period are expected to be small and noisy (Supplementary Figure S9) due to biological variability, multisite scanner drifting, or related technical sources, suggesting the changes in the SUVR trajectories reflect spurious change. A linear SUVR time course over 1.5 years with a maximum of three time points is a reasonable assumption (Supplementary Figure S11); longer observation periods are required to model SUVR using more complex models.

The dissimilarities in regional SUVR change between the two studies in participants with prodromal to mild AD may be attributed to the NHS's smaller sample size and the cognitive status differences. The NHS prodromal participants exhibited overall less cognitive impairment alongside a lower tau burden associated with a higher accumulation over time, particularly in the MT region. A ceiling effect and longitudinal decreases were observed in participants with higher baseline tau burden. The analysis by tau burden level also showed regional differences between the groups. The Tauriel low-tau group showed the lowest tau accumulation in all regions except the MT. The accumulation in MT may be associated with aging and possible additional non-AD pathologies. Overall, we observed larger increases in tau accumulation in the mid- to high-tau-level group (including the NHS moderate and Tauriel mild AD participants) with the WCG and mTMP capturing the overall global change while other cortical subregions can provide additional regional information.

### 4.1 Conclusion

Although PVC may be an acceptable alternative for addressing the impact of atrophy on tau PET measurements, its implementation can be challenging due to the wide range of image characteristics and spatial resolutions in multisite imaging studies. The evaluation of various commonly used PET PVC methods in a relatively modest-size study showed the additional variability introduced by these methods may hinder accurate the evaluation of longitudinal tau burden. Our results support the estimation of the SUVR change from the LMEM slope analysis. LMEM offers a robust framework for the quantification of longitudinal tau PET with low longitudinal test–retest variability. This approach can help address issues with missing data or variable intervals between visits due to missing imaging appointments or operational challenges in larger clinical trials.

## Data availability statement

Qualified researchers may request access to individual patient-level data through the clinical study data request platform (https://vivli.org/). Further details on Roche's criteria for eligible studies are available here: https://vivli.org/members/ourmembers/. For further details on Roche's Global Policy on the Sharing of Clinical Information and how to request access to related clinical study documents, see https://www.roche.com/innovation/process/clinical-trials/data-sharing/.

## Ethics statement

The studies involving humans were approved by the each research center's Institutional Review Board prior to commencing recruitment and was conducted in accordance with the International Conference on Harmonization E6 Guidelines for Good Clinical Practice. Written informed consent was obtained for all participants and/or their legally authorized representatives prior to performing study-related procedures in accordance with federal and institutional guidelines. The studies were conducted in accordance with the local legislation and institutional requirements.

## Author contributions

SSB: Conceptualization, Data curation, Formal analysis, Writing—original draft, Writing—review & editing. SB: Data curation, Formal analysis, Writing—review & editing. PM: Conceptualization, Data curation, Writing—review & editing. MT: Data curation, Formal analysis, Writing—review & editing. CG: Data curation, Formal analysis, Writing—review & editing. KW: Conceptualization, Data curation, Writing—review & editing. YZ: Data curation, Formal analysis, Writing—review & editing. GK: Conceptualization, Data curation, Writing—review & editing. RW: Data curation, Writing—review & editing. ET: Conceptualization, Data curation, Writing—review & editing.
