# Supplementary material for: Evaluation of partial volume correction and analysis of longitudinal [18F]GTP1 tau PET imaging in Alzheimer's disease using linear mixed-effects models
Source: Front Neuroimaging. 2024 Mar 28;3:1355402. doi: 10.3389/fnimg.2024.1355402 (PMC11008283; doi:10.3389/fnimg.2024.1355402)
Supplement: Supplementary file 1 [file Data_Sheet_1.PDF]

Supplementary Materials

Frontiers in Neuroimaging

Evaluation of partial volume correction and analysis of longitudinal [^{18}F]GTP1 tau PET imaging using linear mixed-effects models in Alzheimer's disease

Sandra M. Sanabria Bohórquez[a]; Suzanne Baker[a, b]; Paul T. Manser[c]; Matteo Tonietto[d], Christopher Galli[d], Kristin R. Wildsmith[e]; Yixuan Zou[f]; Geoffrey A. Kerchner[d]; Robby Weimer[g]; Edmond Teng[h]

[a]Clinical Imaging Group, Genentech, Inc., South San Francisco, CA, USA
[b]Molecular Biophysics and Integrated Bioimaging, Lawrence Berkeley National Laboratory, Berkeley, CA, USA
[c]Biostatistics, Genentech, Inc., South San Francisco, CA, USA
[d]Roche Pharma Research and Early Development, Roche Innovation Center Basel, F. Hoffmann-La Roche Ltd., Basel, Switzerland
[e]Biomarker Development, Genentech, Inc., South San Francisco, CA, USA
[f]Data and Statistical Science, Product Development, Hoffmann-La Roche Ltd., Basel, Switzerland
[g]Translational Imaging, Genentech, Inc., South San Francisco, CA, USA
[h]Early Clinical Development, Genentech, Inc., South San Francisco, CA, USA

Address for Correspondence:
Sandra M. Sanabria Bohórquez, PhD
Genentech, Inc.
1 DNA Way
South San Francisco, CA 94080, USA
Tel: 650-467-5242
Email: sanabria.sandra@gene.com

ORCiD numbers:
Sandra M. Sanabria Bohórquez: https://orcid.org/0000-0003-2890-2076
Geoffrey A. Kerchner: https://orcid.org/0000-0001-7674-0695
Matteo Tonietto: https://orcid.org/0000-0001-9591-5710
Robby M Weimer: https://orcid.org/0000-0001-8308-2651
Yixuan Zou: https://orcid.org/0000-0003-2477-6906
Suzanne Baker: https://orcid.org/0000-0003-0209-3127
Edmond Teng: https://orcid.org/0000-0002-1375-857X

Table S1. Longitudinal group effect size or *t* scores of data not corrected for partial volume

	***t* score (group separation)**			***p* value**		
**Region**	**Low tau vs CU**	**Mid tau vs CU**	**High tau vs CU**	**Low tau vs CU**	**Mod tau vs CU**	**High tau vs CU**
MT	1.93	2.02	0.56	0.054	0.045	0.575
mTMP	1.84	3.04	2.35	0.067	0.003	0.020
Rest of temporal	2.29	3.46	2.70	0.023	0.001	0.007
Parietal	1.82	3.55	3.25	0.069	0.000	0.001
Occipital	2.05	2.57	2.90	0.042	0.011	0.004
Frontal	1.84	3.01	3.15	0.068	0.003	0.002
TMP	1.91	2.92	2.09	0.058	0.004	0.037
WCG	2.04	3.25	2.98	0.043	0.001	0.003
	**t-score (group separation)**			**p-value**		
**Region**	**Prodromal vs CU**	**Mild vs CU**	**Moderate vs CU**	**Prodromal vs CU**	**Mild vs CU**	**Moderate vs CU**
MTL	2.63	1.03	0.70	0.009	0.305	0.488
mTMP	2.81	2.09	2.64	0.005	0.038	0.009
Rest of temporal	3.34	2.28	2.91	0.001	0.023	0.004
Parietal	2.79	2.61	3.53	0.006	0.010	0.001
Occipital	2.49	1.86	3.17	0.013	0.065	0.002
Frontal	2.70	2.14	3.20	0.008	0.033	0.002
TMP	2.83	1.99	2.34	0.005	0.048	0.020
WCG	2.96	2.27	3.23	0.003	0.024	0.001

CU, cognitively unimpaired; Mod, moderate; MT, mesial temporal entorhinal cortex, hippocampus, and amygdala; mTMP, TMP excluding MT; TMP, temporal meta-ROI; WCG, whole cortical gray.

Table S2. Within-subject percentage variability of longitudinal [^{18}F]GTP1 SUVR measurements in non-PVC and PVC data. Data are expressed as % (95% CI).

Region	Non-PVC	PVC-2 compartments	PVC-3 compartments	PVC-VC15	PVC-RBV
MT	4.2 (3.6, 4.8)%	4.2 (3.7, 4.8)%	4.7 (4.1, 5.4)%	4.7 (4.1, 5.3)%	6.5 (5.6, 7.4)%
mTMP	3.2 (2.8, 3.7)%	3.3 (3.0, 3.8)%	3.6 (3.1, 4.0)%	3.5 (3.1, 4.0)%	4.6 (4.1, 5.3)%
Rest of temporal	3.2 (2.8, 3.6)%	3.4 (3.0, 3.8)%	3.7 (3.2, 4.1)%	3.3 (2.9, 3.8)%	4.2 (3.7, 4.8)%
Parietal	3.4 (3.0, 3.9)%	3.7 (3.3, 4.2)%	4.1 (3.6, 4.6)%	3.6 (3.2, 4.1)%	4.9 (4.3, 5.6)%
Occipital	3.9 (3.4, 4.4)%	4.3 (3.8, 4.8)%	4.9 (4.3, 5.5)%	4.2 (3.7, 4.8)%	6.3 (5.5, 7.1)%
Frontal	3.0 (2.7, 3.4)%	3.2 (2.8, 3.7)%	3.5 (3.1, 4.0)%	3.1 (2.7, 3.5)%	4.1 (3.6, 4.7)%
TMP	3.3 (2.9, 3.7)%	3.4 (3.0, 3.8)%	3.6 (3.2, 4.1)%	3.6 (3.1, 4.0)%	4.7 (4.1, 5.3)%
WCG	3.0 (2.7, 3.4)%	3.2 (2.8, 3.6)%	3.5 (3.1, 4.0)%	3.2 (2.8, 3.6)%	4.0 (3.5, 4.5)%

Comp, compartment; MT, mesial temporal entorhinal cortex, hippocampus, and amygdala; mTMP, TMP excluding MT; PVC, partial volume correction; RBV, region-based voxelwise method; SUVR, standardized uptake value ratio; TMP, temporal meta-ROI; VC, van Cittert iterative deconvolution; WCG, whole cortical gray.

Table S3. Natural history study: average annualized percentage change in SUVR (without partial volume correction) estimated at weeks 26, 52, and 78, and the corresponding effect size calculated as the ratio of the mean and the standard deviation. Average and 95% CIs were estimated applying bootstrap.

Region	Week 26 %ΔSUVR/year (95% CI)							
	**CU**	**Prodromal AD**	**Mild AD**	**Moderate AD**	**CU**	**Low tau**	**Mid tau**	**High tau**
	***n* = 10**	***n* = 24**	***n* = 15**	***n* = 14**	***n* = 10**	***n* = 13**	***n* = 30**	***n* = 10**
MT	-1.18 (-9.77, 6.77)	4.87 (0.25, 9.02)	-2.61 (-8.16, 2.80)	0.92 (-6.96, 7.57)	-1.18 (-9.77, 6.77)	3.69 (-5.95, 10.19)	1.66 (-2.09, 5.33)	-0.68 (-8.33, 8.90)
mTMP	-2.88 (-7.81, 2.56)	3.34 (0.82, 6.41)	-2.11 (-7.06, 2.58)	1.06 (-5.78, 6.76)	-2.88 (-7.81, 2.56)	4.22 (-2.51, 8.45)	0.64 (-2.17, 3.59)	-1.01 (-6.44, 6.43)
Rest of temporal	-5.88 (-10.69, -0.55)	1.84 (-0.64, 4.73)	-2.53 (-8.95, 1.47)	2.57 (-3.31, 8.75)	-5.88 (-10.69, -0.55)	3.19 (-1.18, 7.45)	0.18 (-3.13, 3.13)	-0.34 (-5.94, 9.91)
Parietal	-4.51 (-10.45, 1.85)	2.33 (-0.84, 4.97)	-2.90 (-9.07, 0.50)	3.88 (-1.01, 10.89)	-4.51 (-10.45, 1.85)	4.35 (0.26, 7.97)	0.24 (-3.27, 3.28)	0.25 (-4.34, 8.92)
Occipital	-5.10 (-10.67, 2.07)	5.83 (1.51, 9.71)	-2.40 (-7.76, 3.74)	4.33 (-2.66, 10.92)	-5.10 (-10.67, 2.07)	7.53 (0.85, 14.42)	1.69 (-1.80, 5.02)	1.45 (-6.98, 12.00)
Frontal	-5.89 (-13.49, 0.36)	1.18 (-1.57, 3.90)	-1.76 (-6.40, 1.81)	3.32 (-2.61, 9.65)	-5.89 (-13.49, 0.36)	2.55 (-1.97, 6.70)	-0.07 (-3.20, 3.59)	1.81 (-3.60, 7.52)
TMP	-2.77 (-7.85, 3.14)	3.64 (0.83, 6.96)	-2.03 (-7.18, 2.29)	0.93 (-5.70, 6.54)	-2.77 (-7.85, 3.14)	4.10 (-3.62, 9.48)	0.88 (-1.92, 3.88)	-1.05 (-7.02, 6.26)
WCG	-4.76 (-9.87, 1.22)	2.61 (-0.15, 5.26)	-2.45 (-7.40, 0.99)	2.78 (-2.36, 9.05)	-4.76 (-9.87, 1.22)	4.00 (-0.73, 8.11)	0.35 (-2.52, 3.28)	0.27 (-4.41, 8.44)

Region	Week 26 %ΔSUVR/year Effect Size (95% CI)							
	**CU**	**Prodromal AD**	**Mild AD**	**Moderate AD**	**CU**	**Low tau**	**Mid tau**	**High tau**
	***n* = 10**	***n* = 24**	***n* = 15**	***n* = 14**	***n* = 10**	***n* = 13**	***n* = 30**	***n* = 10**
MT	-0.09 (-0.89, 0.66)	0.45 (-0.03, 0.95)	-0.24 (-0.80, 0.33)	0.06 (-0.56, 0.68)	-0.09 (-0.89, 0.66)	0.27 (-0.40, 1.10)	0.15 (-0.24, 0.54)	-0.05 (-0.89, 0.78)
mTMP	-0.36 (-1.25, 0.52)	0.47 (0.01, 0.91)	-0.22 (-0.86, 0.37)	0.09 (-0.51, 0.76)	-0.36 (-1.25, 0.52)	0.39 (-0.38, 1.23)	0.09 (-0.29, 0.49)	-0.10 (-0.94, 0.71)
Rest of temporal	-0.73 (-1.53, 0.28)	0.28 (-0.15, 0.69)	-0.24 (-0.77, 0.35)	0.22 (-0.35, 0.82)	-0.73 (-1.53, 0.28)	0.40 (-0.30, 1.06)	0.03 (-0.35, 0.40)	-0.04 (-1.02, 0.87)
Parietal	-0.48 (-1.49, 0.32)	0.32 (-0.14, 0.73)	-0.34 (-0.78, 0.21)	0.36 (-0.22, 0.87)	-0.48 (-1.49, 0.32)	0.61 (0.04, 1.29)	0.02 (-0.35, 0.39)	0.02 (-1.03, 0.88)
Occipital	-0.50 (-1.50, 0.36)	0.57 (0.10, 1.03)	-0.20 (-0.87, 0.41)	0.35 (-0.34, 1.01)	-0.50 (-1.50, 0.36)	0.61 (-0.05, 1.35)	0.18 (-0.21, 0.57)	0.10 (-0.76, 0.91)
Frontal	-0.52 (-1.12, 0.65)	0.19 (-0.24, 0.61)	-0.22 (-0.74, 0.37)	0.28 (-0.29, 0.86)	-0.52 (-1.12, 0.65)	0.33 (-0.39, 1.01)	0.00 (-0.39, 0.37)	0.19 (-0.65, 0.91)
TMP	-0.29 (-1.16, 0.60)	0.50 (-0.01, 0.95)	-0.23 (-0.86, 0.33)	0.08 (-0.55, 0.72)	-0.29 (-1.16, 0.60)	0.37 (-0.44, 1.18)	0.11 (-0.29, 0.49)	-0.10 (-0.83, 0.79)
WCG	-0.52 (-1.27, 0.37)	0.38 (-0.03, 0.79)	-0.30 (-0.85, 0.21)	0.27 (-0.34, 0.85)	-0.52 (-1.27, 0.37)	0.50 (-0.16, 1.20)	0.05 (-0.35, 0.41)	0.03 (-0.88, 0.79)

Region	Week 52 %ΔSUVR/year (95% CI)							
	**CU**	**Prodromal AD**	**Mild AD**	**Moderate AD**	**CU**	**Low tau**	**Mid tau**	**High tau**
	***n* = 9**	***n* = 20**	***n* = 15**	***n* = 12**	***n* = 9**	***n* = 12**	***n* = 26**	***n* = 9**
MT	-2.44 (-8.61, 1.64)	3.99 (1.42, 6.44)	0.05 (-3.86, 4.62)	-1.53 (-5.07, 1.90)	-2.44 (-8.61, 1.64)	1.61 (-2.82, 5.63)	1.83 (-0.90, 4.66)	-0.47 (-4.72, 4.28)
mTMP	-2.44 (-6.39, 1.61)	3.93 (2.28, 6.09)	2.37 (-0.95, 7.28)	1.94 (-1.40, 4.45)	-2.44 (-6.39, 1.61)	1.86 (-1.46, 4.33)	3.85 (1.50, 6.40)	1.62 (-1.26, 4.79)
Rest of temporal	-2.93 (-7.55, 0.71)	2.74 (1.10, 5.18)	0.97 (-1.77, 4.33)	1.45 (-1.79, 4.85)	-2.93 (-7.55, 0.71)	0.92 (-1.87, 3.40)	2.52 (0.32, 4.84)	1.05 (-1.92, 5.54)
Parietal	-1.76 (-5.62, 3.74)	3.44 (1.28, 6.45)	2.31 (-0.99, 4.46)	3.68 (1.42, 6.02)	-1.76 (-5.62, 3.74)	1.99 (-0.30, 4.57)	3.81 (1.44, 6.31)	2.74 (0.63, 5.03)
Occipital	-1.17 (-6.00, 6.83)	3.97 (1.70, 7.13)	0.84 (-1.81, 3.53)	2.66 (-1.34, 5.96)	-1.17 (-6.00, 6.83)	2.79 (0.05, 7.92)	2.63 (0.28, 4.86)	2.48 (-1.56, 5.94)
Frontal	-2.79 (-7.80, 1.24)	2.07 (-0.40, 4.30)	0.94 (-1.73, 3.51)	2.44 (-0.46, 5.32)	-2.79 (-7.80, 1.24)	0.87 (-2.04, 2.92)	2.02 (-0.15, 4.32)	2.42 (-0.35, 4.97)
TMP	-2.53 (-6.85, 0.78)	3.80 (2.20, 6.17)	2.06 (-0.93, 6.97)	1.20 (-2.26, 4.08)	-2.53 (-6.85, 0.78)	1.76 (-1.77, 4.61)	3.43 (1.27, 6.17)	1.25 (-1.72, 5.16)
WCG	-2.34 (-6.38, 2.03)	2.99 (1.32, 5.89)	1.37 (-1.58, 4.17)	2.31 (-0.35, 4.98)	-2.34 (-6.38, 2.03)	1.59 (-0.73, 4.09)	2.78 (0.63, 4.95)	1.90 (-0.41, 4.79)

Region	Week 52 %ΔSUVR/year effect size (95% CI)							
	**CU**	**Prodromal AD**	**Mild AD**	**Moderate AD**	**CU**	**Low tau**	**Mid tau**	**High tau**
	***n* = 9**	***n* = 20**	***n* = 15**	***n* = 12**	***n* = 9**	***n* = 12**	***n* = 26**	***n* = 9**
MT	-0.33 (-1.15, 0.62)	0.69 (0.17, 1.14)	0.00 (-0.59, 0.59)	-0.23 (-0.93, 0.42)	-0.33 (-1.15, 0.62)	0.24 (-0.49, 0.88)	0.25 (-0.18, 0.63)	-0.06 (-0.92, 0.99)
mTMP	-0.44 (-1.11, 0.62)	0.88 (0.50, 1.27)	0.31 (-0.29, 0.78)	0.35 (-0.43, 1.09)	-0.44 (-1.11, 0.62)	0.36 (-0.48, 1.08)	0.59 (0.13, 0.93)	0.34 (-0.46, 1.19)
Rest of temporal	-0.48 (-1.30, 0.32)	0.60 (0.12, 0.97)	0.16 (-0.46, 0.68)	0.24 (-0.41, 0.92)	-0.48 (-1.30, 0.32)	0.20 (-0.50, 0.99)	0.43 (-0.02, 0.81)	0.21 (-0.63, 1.07)
Parietal	-0.26 (-1.21, 0.79)	0.58 (0.10, 0.95)	0.41 (-0.41, 1.15)	0.91 (0.35, 1.61)	-0.26 (-1.21, 0.79)	0.44 (-0.40, 1.03)	0.62 (0.08, 1.04)	0.82 (0.40, 1.54)
Occipital	-0.14 (-1.05, 1.09)	0.69 (0.26, 1.09)	0.17 (-0.45, 0.72)	0.41 (-0.31, 1.15)	-0.14 (-1.05, 1.09)	0.45 (-0.34, 0.92)	0.45 (0.05, 0.88)	0.42 (-0.54, 1.36)
Frontal	-0.40 (-1.28, 0.43)	0.40 (-0.10, 0.84)	0.17 (-0.47, 0.72)	0.48 (-0.24, 1.26)	-0.40 (-1.28, 0.43)	0.20 (-0.47, 1.08)	0.34 (-0.07, 0.73)	0.60 (-0.26, 1.77)
TMP	-0.43 (-1.14, 0.58)	0.87 (0.49, 1.26)	0.26 (-0.30, 0.79)	0.23 (-0.52, 0.91)	-0.43 (-1.14, 0.58)	0.34 (-0.48, 1.07)	0.54 (0.10, 0.90)	0.25 (-0.57, 1.13)
WCG	-0.36 (-1.22, 0.68)	0.63 (0.19, 1.01)	0.24 (-0.40, 0.77)	0.50 (-0.25, 1.25)	-0.36 (-1.22, 0.68)	0.37 (-0.36, 1.05)	0.50 (0.07, 0.88)	0.50 (-0.28, 1.45)

Region	Week 78 %ΔSUVR/year (95% CI)							
	**CU**	**Prodromal AD**	**Mild AD**	**Moderate AD**	**CU**	**Low tau**	**Mid tau**	**High tau**
	***n* = 9**	***n* = 21**	***n* = 10**	***n* = 11**	***n* = 9**	***n* = 12**	***n* = 21**	***n* = 9**
MT	-1.66 (-5.16, 1.94)	2.57 (1.17, 4.01)	0.04 (-2.53, 3.49)	-0.43 (-3.65, 1.95)	-1.66 (-5.16, 1.94)	2.05 (-0.55, 4.56)	1.43 (-0.09, 3.13)	-0.58 (-4.31, 2.24)
mTMP	-0.96 (-2.93, 1.02)	3.08 (1.94, 4.38)	1.84 (-1.17, 5.48)	3.00 (0.54, 5.13)	-0.96 (-2.93, 1.02)	2.22 (0.32, 4.69)	2.99 (1.74, 4.21)	2.95 (-1.35, 6.31)
Rest of temporal	-2.63 (-4.92, -0.74)	1.88 (0.88, 3.17)	0.68 (-1.73, 4.68)	1.32 (-0.71, 3.41)	-2.63 (-4.92, -0.74)	0.99 (-0.40, 2.69)	1.71 (0.25, 3.23)	1.46 (-1.18, 3.82)
Parietal	-2.05 (-3.78, -0.41)	1.91 (0.57, 3.30)	1.84 (-0.92, 5.97)	3.48 (1.05, 5.18)	-2.05 (-3.78, -0.41)	0.98 (-0.36, 2.44)	2.66 (0.90, 4.61)	3.24 (-0.45, 5.55)
Occipital	-1.69 (-3.54, 0.06)	2.80 (1.46, 4.30)	2.37 (-0.05, 6.01)	4.33 (0.97, 7.86)	-1.69 (-3.54, 0.06)	2.81 (1.10, 4.97)	2.78 (1.33, 4.50)	4.18 (-0.18, 8.50)
Frontal	-2.61 (-6.14, -0.27)	1.26 (0.20, 2.79)	1.07 (-1.69, 4.52)	2.21 (-1.03, 4.21)	-2.61 (-6.14, -0.27)	0.41 (-1.15, 2.02)	1.55 (-0.02, 3.41)	2.64 (0.14, 4.91)
TMP	-1.11 (-3.59, 1.23)	2.99 (1.83, 4.33)	1.59 (-1.26, 5.22)	2.39 (-0.53, 4.26)	-1.11 (-3.59, 1.23)	2.22 (0.07, 4.44)	2.71 (1.43, 3.88)	2.42 (-1.94, 5.64)
WCG	-2.06 (-4.30, -0.07)	1.98 (0.96, 3.13)	1.46 (-1.55, 5.00)	2.58 (-0.08, 4.30)	-2.06 (-4.30, -0.07)	1.32 (-0.16, 3.00)	2.14 (0.76, 3.73)	2.63 (-0.40, 5.01)

Region	Week 78 %ΔSUVR/year effect size (95% CI)							
	**CU**	**Prodromal AD**	**Mild AD**	**Moderate AD**	**CU**	**Low tau**	**Mid tau**	**High tau**
	***n* = 9**	***n* = 21**	***n* = 10**	***n* = 11**	***n* = 9**	***n* = 12**	***n* = 21**	***n* = 9**
MT	-0.29 (-1.11, 0.80)	0.76 (0.33, 1.23)	0.02 (-0.80, 0.83)	-0.09 (-0.82, 0.62)	-0.29 (-1.11, 0.80)	0.44 (-0.25, 1.14)	0.37 (-0.09, 0.85)	-0.12 (-0.97, 0.78)
mTMP	-0.31 (-1.12, 0.64)	1.07 (0.61, 1.51)	0.35 (-0.43, 1.12)	0.79 (-0.05, 1.99)	-0.31 (-1.12, 0.64)	0.62 (-0.01, 1.17)	0.97 (0.39, 1.48)	0.54 (-0.23, 2.51)
Rest of temporal	-0.83 (-1.64, -0.03)	0.74 (0.21, 1.11)	0.14 (-0.72, 0.94)	0.38 (-0.39, 1.13)	-0.83 (-1.64, -0.03)	0.35 (-0.41, 0.87)	0.50 (-0.02, 0.94)	0.36 (-0.48, 1.45)
Parietal	-0.83 (-1.62, 0.09)	0.62 (0.15, 1.01)	0.33 (-0.49, 0.98)	0.99 (0.21, 2.07)	-0.83 (-1.62, 0.09)	0.39 (-0.28, 0.99)	0.61 (0.06, 1.05)	0.75 (-0.14, 1.94)
Occipital	-0.62 (-1.75, 0.31)	0.87 (0.44, 1.36)	0.48 (-0.31, 1.10)	0.76 (-0.09, 1.42)	-0.62 (-1.75, 0.31)	0.84 (0.30, 1.45)	0.74 (0.26, 1.18)	0.64 (-0.33, 1.47)
Frontal	-0.62 (-1.23, 0.34)	0.44 (-0.15, 0.77)	0.23 (-0.62, 0.96)	0.54 (-0.38, 1.48)	-0.62 (-1.23, 0.34)	0.15 (-0.66, 0.80)	0.40 (-0.11, 0.83)	0.73 (-0.22, 1.58)
TMP	-0.31 (-1.13, 0.67)	1.05 (0.59, 1.46)	0.30 (-0.45, 1.11)	0.62 (-0.18, 1.78)	-0.31 (-1.13, 0.67)	0.60 (-0.10, 1.15)	0.86 (0.31, 1.34)	0.44 (-0.39, 1.77)
WCG	-0.63 (-1.40, 0.23)	0.75 (0.31, 1.13)	0.28 (-0.55, 0.95)	0.76 (-0.05, 2.58)	-0.63 (-1.40, 0.23)	0.45 (-0.25, 1.05)	0.60 (0.07, 1.01)	0.64 (-0.19, 2.23)

AD, Alzheimer's disease; CI, confidence interval; CU, cognitively unimpaired; MT, mesial temporal entorhinal cortex, hippocampus, and amygdala; mTMP, TMP excluding MT; ROI, region of interest; SUVR, standardized uptake value ratio; TMP, temporal meta-ROI; WCG, whole cortical gray.

Table S4. Tauriel study: average annualized percentage change in SUVR relative, baseline at each follow up visit and the corresponding effect size calculated as the ratio of the mean and the standard deviation. Average and 95% CIs were estimated applying bootstrap.

Region	Visit	%ΔSUVR/year (95% CI)				
		Prodromal AD	Mild AD	Low tau	Mid tau	High tau
Number of subjects	W49	**113**	201	**78**	**156**	**80**
	W73	109	**178**	**71**	**147**	**69**
MT	W49	1.27 (-0.14, 2.44)	2.03 (1.17, 2.97)	2.12 (0.94, 3.63)	2.05 (0.87, 3.10)	0.78 (-0.50, 2.43)
	W73	0.83 (-0.11, 1.60)	0.75 (0.18, 1.39)	1.39 (0.53, 2.24)	1.09 (0.32, 1.90)	-0.56 (-1.43, 0.33)
mTMP	W49	3.69 (2.51, 4.84)	5.00 (4.10, 5.96)	2.86 (1.89, 4.56)	5.46 (4.38, 6.47)	4.22 (2.72, 5.92)
	W73	5.06 (3.47, 6.25)	5.56 (4.60, 6.60)	3.76 (2.48, 5.04)	7.09 (5.93, 8.38)	3.44 (1.94, 5.06)
Rest of temporal	W49	2.80 (1.61, 3.91)	4.52 (3.59, 5.45)	1.76 (0.76, 3.15)	4.61 (3.48, 5.50)	4.58 (2.93, 6.09)
	W73	3.67 (2.28, 4.95)	4.82 (3.87, 5.88)	2.29 (1.07, 3.53)	5.41 (4.19, 6.71)	4.32 (2.62, 6.12)
Parietal	W49	3.41 (1.98, 4.62)	4.21 (3.30, 5.22)	2.18 (1.13, 3.33)	4.50 (3.41, 5.61)	4.50 (2.89, 6.14)
	W73	4.33 (2.78, 5.74)	4.74 (3.65, 5.94)	3.10 (1.72, 4.61)	5.52 (4.14, 6.86)	4.05 (2.25, 6.01)
Occipital	W49	2.43 (1.48, 3.39)	3.40 (2.69, 4.09)	1.99 (1.07, 3.49)	2.82 (1.98, 3.58)	4.56 (3.32, 5.71)
	W73	3.24 (2.04, 4.40)	3.80 (2.98, 4.64)	2.09 (1.09, 3.24)	4.00 (2.80, 4.94)	4.28 (2.85, 5.86)
Frontal	W49	2.75 (1.50, 3.94)	4.45 (3.54, 5.31)	1.74 (0.77, 3.48)	4.27 (3.22, 5.38)	4.95 (3.41, 6.39)
	W73	3.68 (2.31, 5.19)	4.93 (3.73, 5.98)	2.26 (1.00, 3.77)	5.30 (3.85, 6.66)	4.97 (3.32, 6.93)
TMP	W49	3.30 (1.85, 4.44)	4.66 (3.90, 5.69)	2.80 (1.84, 4.78)	5.02 (3.92, 6.12)	3.96 (2.30, 5.61)
	W73	3.04 (2.16, 3.88)	3.28 (2.58, 3.95)	2.35 (1.49, 3.21)	4.20 (3.43, 5.00)	1.90 (0.94, 3.02)
WCG	W49	2.87 (1.74, 3.96)	4.27 (3.53, 5.17)	2.09 (1.10, 3.53)	4.24 (3.21, 5.28)	4.40 (2.89, 5.84)
	W73	2.60 (1.50, 3.51)	3.05 (2.38, 3.79)	1.78 (1.00, 2.67)	3.56 (2.72, 4.35)	2.58 (1.48, 3.74)

Region	Visit	Effect Size (95% CI)				
		Prodromal AD	Mild AD	Low tau	Mid tau	High tau
MT	W49	0.18 (-0.05, 0.37)	0.32 (0.18, 0.45)	0.35 (0.14, 0.53)	0.31 (0.10, 0.47)	0.12 (-0.10, 0.34)
	W73	0.18 (-0.05, 0.36)	0.17 (0.03, 0.32)	0.37 (0.13, 0.60)	0.23 (0.05, 0.39)	-0.14 (-0.39, 0.09)
mTMP	W49	0.57 (0.26, 0.77)	0.73 (0.60, 0.86)	0.49 (0.30, 0.66)	0.84 (0.52, 1.02)	0.57 (0.34, 0.79)
	W73	0.67 (0.31, 0.90)	0.81 (0.64, 0.96)	0.70 (0.48, 0.91)	0.92 (0.57, 1.14)	0.51 (0.26, 0.77)
Rest of temporal	W49	0.45 (0.18, 0.65)	0.68 (0.54, 0.80)	0.34 (0.15, 0.51)	0.72 (0.46, 0.88)	0.62 (0.37, 0.86)
	W73	0.49 (0.19, 0.72)	0.67 (0.53, 0.81)	0.44 (0.18, 0.66)	0.71 (0.38, 0.90)	0.56 (0.33, 0.80)
Parietal	W49	0.51 (0.29, 0.69)	0.59 (0.46, 0.72)	0.46 (0.25, 0.65)	0.61 (0.43, 0.76)	0.59 (0.36, 0.83)
	W73	0.52 (0.22, 0.72)	0.60 (0.43, 0.75)	0.51 (0.25, 0.73)	0.63 (0.38, 0.80)	0.51 (0.25, 0.77)
Occipital	W49	0.45 (0.17, 0.63)	0.65 (0.53, 0.77)	0.38 (0.21, 0.55)	0.55 (0.29, 0.72)	0.82 (0.64, 1.00)
	W73	0.51 (0.19, 0.72)	0.65 (0.48, 0.80)	0.43 (0.15, 0.67)	0.62 (0.33, 0.81)	0.69 (0.42, 0.94)
Frontal	W49	0.43 (0.20, 0.60)	0.66 (0.53, 0.77)	0.31 (0.12, 0.48)	0.62 (0.42, 0.76)	0.73 (0.47, 0.95)
	W73	0.45 (0.16, 0.63)	0.65 (0.49, 0.80)	0.38 (0.14, 0.61)	0.62 (0.36, 0.79)	0.64 (0.38, 0.89)

TMP	W49	0.51 (0.21, 0.72)	0.68 (0.55, 0.80)	0.47 (0.29, 0.63)	0.75 (0.44, 0.94)	0.53 (0.31, 0.74)
	W73	0.62 (0.28, 0.83)	0.73 (0.58, 0.88)	0.66 (0.42, 0.87)	0.85 (0.52, 1.04)	0.43 (0.19, 0.67)
WCG	W49	0.46 (0.19, 0.65)	0.66 (0.53, 0.78)	0.40 (0.22, 0.59)	0.65 (0.41, 0.81)	0.64 (0.40, 0.87)
	W73	0.52 (0.21, 0.72)	0.65 (0.49, 0.81)	0.50 (0.22, 0.71)	0.68 (0.42, 0.86)	0.55 (0.29, 0.80)

AD, Alzheimer's disease; CI, confidence interval; CU, cognitively unimpaired; MT, mesial temporal entorhinal cortex, hippocampus, and amygdala; mTMP, TMP excluding MT; SUVR, standardized uptake value ratio; TMP, temporal meta-ROI; WCG, whole cortical gray.

Figure S1. Average annualized change in SUVR and the corresponding 95% CI in the Tauriel study estimated from the LMEM slope analysis in the placebo and semorinemab treatment arms in prodromal and mild AD subjects.
AD, Alzheimer's disease; LMEM, linear mixed-effects model; mAD, mild AD; MT, mesial temporal entorhinal cortex, hippocampus, and amygdala; mTMP, TMP excluding MT; pAD, prodromal AD; Pcbo, placebo arm; Semo, semorinemab arm; SUVR, standardized uptake value ratio; WCG, whole cortical gray.

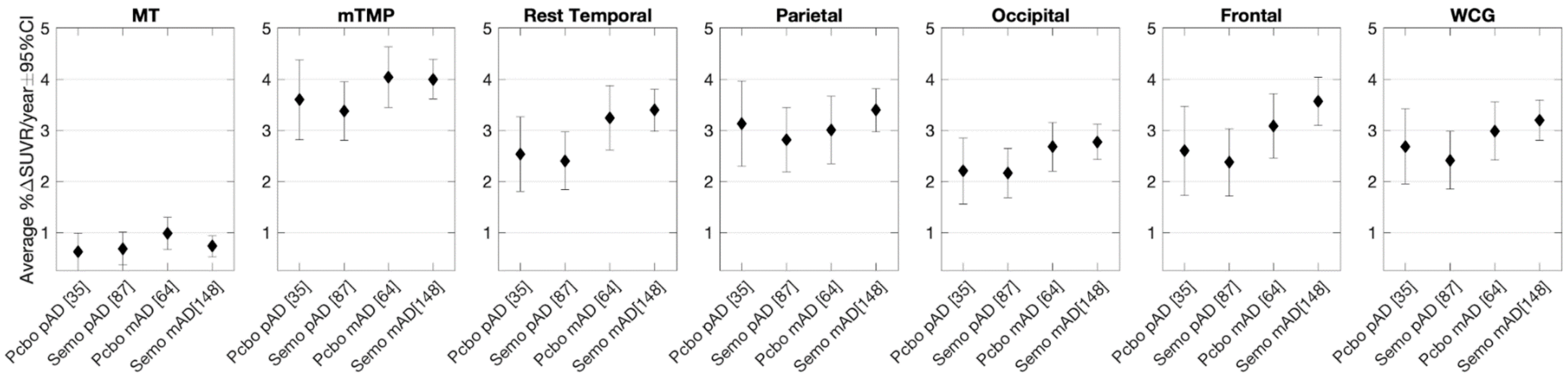

Figure S2. Non-PVC and PVC [^{18}F]GTP1 SUVR versus volume in the TMP. Higher SUVR was associated with decreased cortical volume. PVC could help account for the loss of PET signal in these regions but our data suggest PVC-RBV may be overcorrecting.
CU, cognitively unimpaired; PVC, partial volume correction; PVCVC15, van Cittert iterative deconvolution PVC (alpha = 1.5); PVC2comp and PVC3comp, 2- and 3-compartments PVC; PVCRBV, geometric transfer matrix plus region-based voxelwise PVC; SUVR, standardized uptake value ratio; TMP, temporal meta-region of interest.

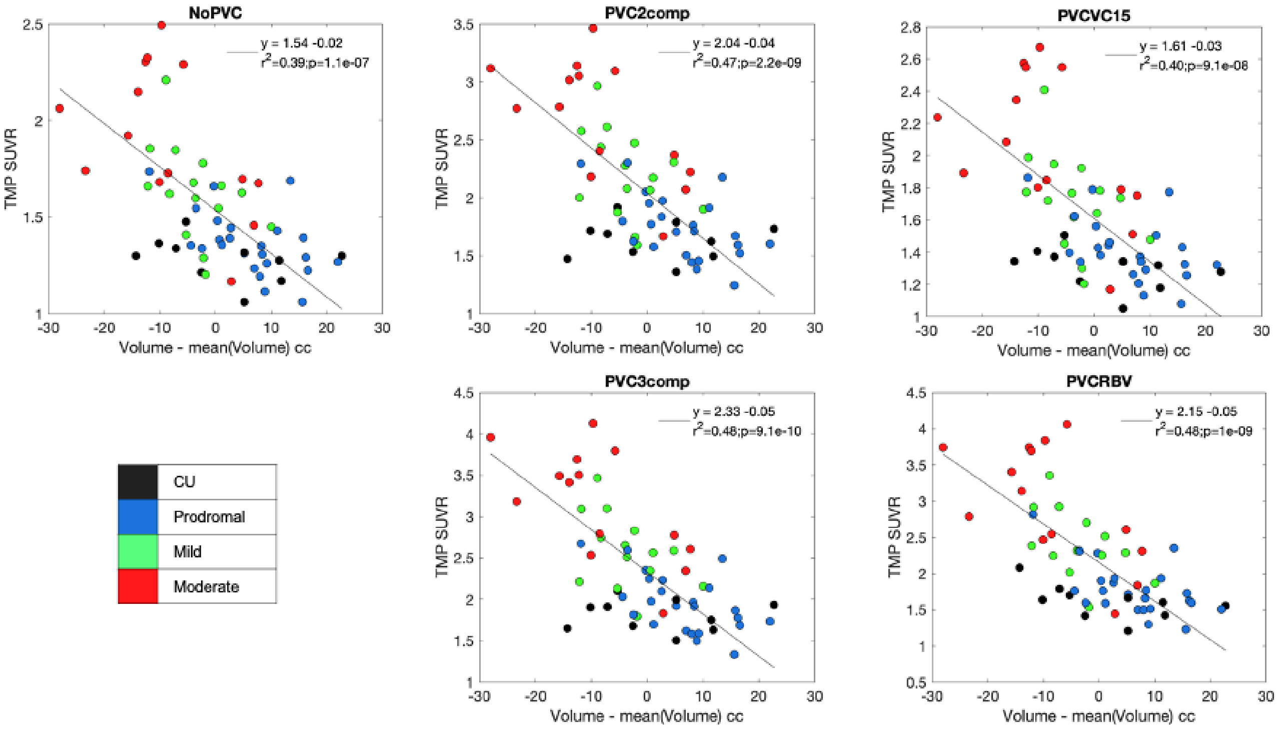

Figure S3. Average baseline SUVR and the corresponding 95% CI in NHS derived from non-PVC and PVC images. Average and 95% confidence intervals were estimated applying bootstrap.
AD, Alzheimer's disease; CI, confidence interval; CU, cognitive unimpaired; Prod, prodromal AD; Mild, mild AD; Mod, moderate AD; MT, mesial temporal entorhinal cortex, hippocampus, and amygdala; mTMP, TMP excluding MT; PVC, Partial volume correction; PVCVC15, van Cittert iterative deconvolution PVC (alpha = 1.5); PVC2comp and PVC3comp, 2- and 3-compartments PVC; PVCRBV, geometric transfer matrix plus region-based voxelwise PVC; SUVR, standardized uptake value ratio; WCG, whole cortical gray.

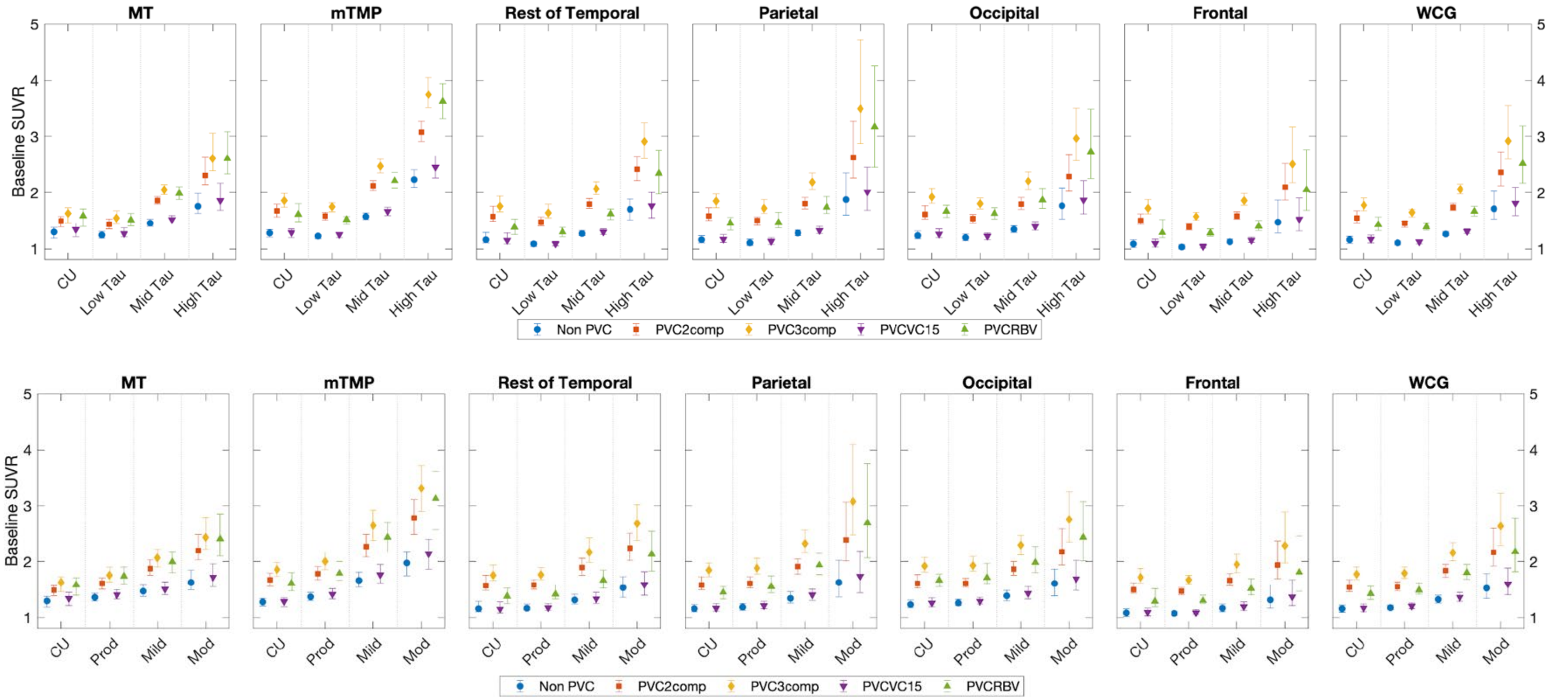

Figure S4. Average annualized percentage change in SUVR and the corresponding 95% CI in NHS participants measured from the LMEM slope analysis without PVC and after applying PVC. The differences between the 2- or 3-compartment and RBV PVC suggest RBV may be overcorrecting regions with higher tau burden. Average and 95% confidence intervals were estimated applying bootstrap.
AD, Alzheimer's disease; CI, confidence interval; CU, cognitive unimpaired; LMEM, linear mixed-effects model; Mild, mild AD; Mod, moderate AD; MT, mesial temporal entorhinal cortex, hippocampus, and amygdala; mTMP, TMP excluding MT; Prod, prodromal AD; PVC, partial volume correction; PVCVC15, van Cittert iterative deconvolution PVC (alpha = 1.5); PVC2comp and PVC3comp, 2- and 3-compartments PVC; PVCRBV, geometric transfer matrix plus region-based voxelwise PVC; RBV, region-based voxelwise; SUVR, standardized uptake value ratio; WCG, whole cortical gray.
.

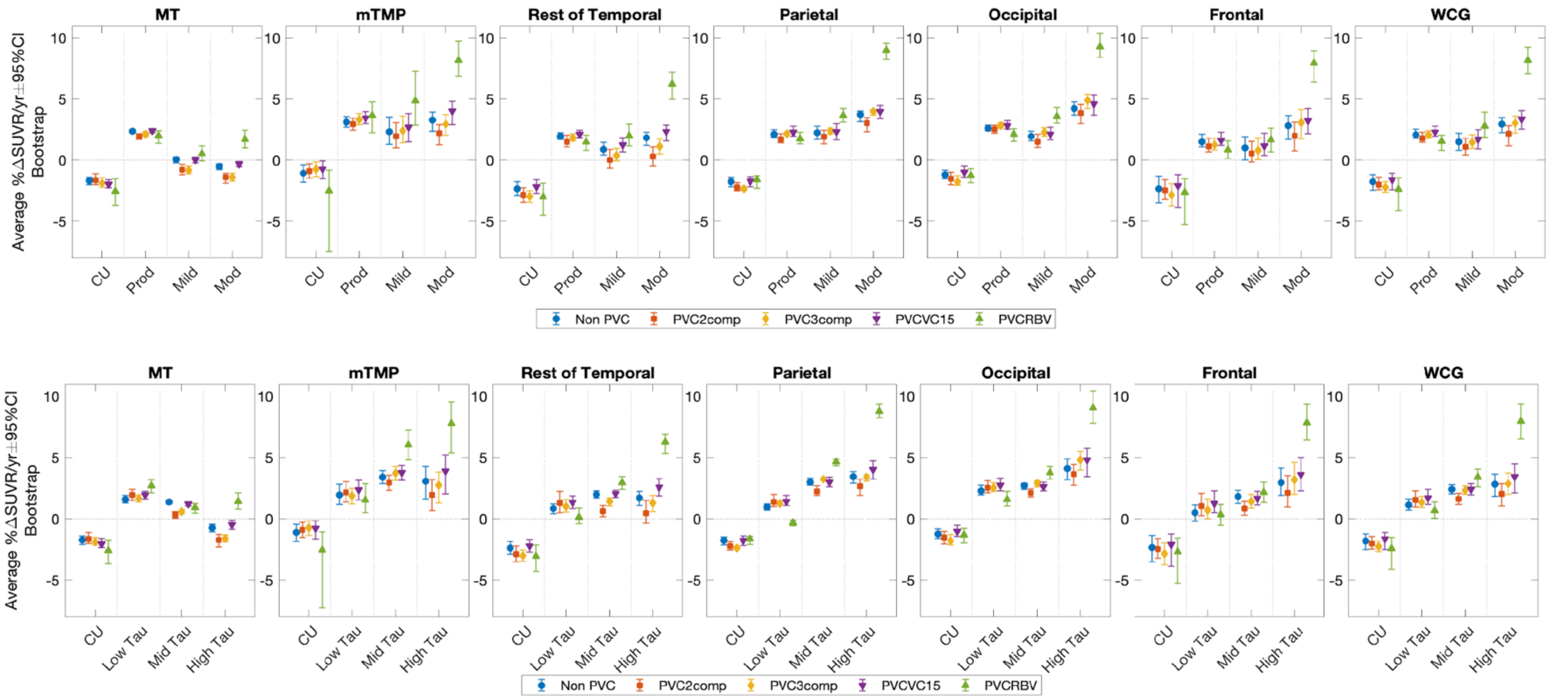

Figure S5. Effect size of average annualized SUVR and the corresponding 95% CI in NHS participants measured from the LMEM slope analysis without PVC and after applying PVC. The differences between the 2- or 3-compartment and RBV PVC suggest RBV may be overcorrecting regions with higher tau burden. Average and 95% confidence intervals were estimated applying bootstrap.

AD, Alzheimer's disease; CU, cognitive unimpaired; LMEM, linear mixed-effects model; MT, mesial temporal entorhinal cortex, hippocampus, and amygdala; mTMP, TMP excluding MT; Prod, prodromal AD; Mild, mild AD; Mod, moderate AD; PVC, partial volume correction; RBV, geometric transfer matrix plus region-based voxelwise PVC; SUVR, standardized uptake value ratio; VC15, van Cittert iterative deconvolution PVC (alpha = 1.5); 2- and 3-comp, 2- and 3-compartments PVC; WCG, whole cortical gray.

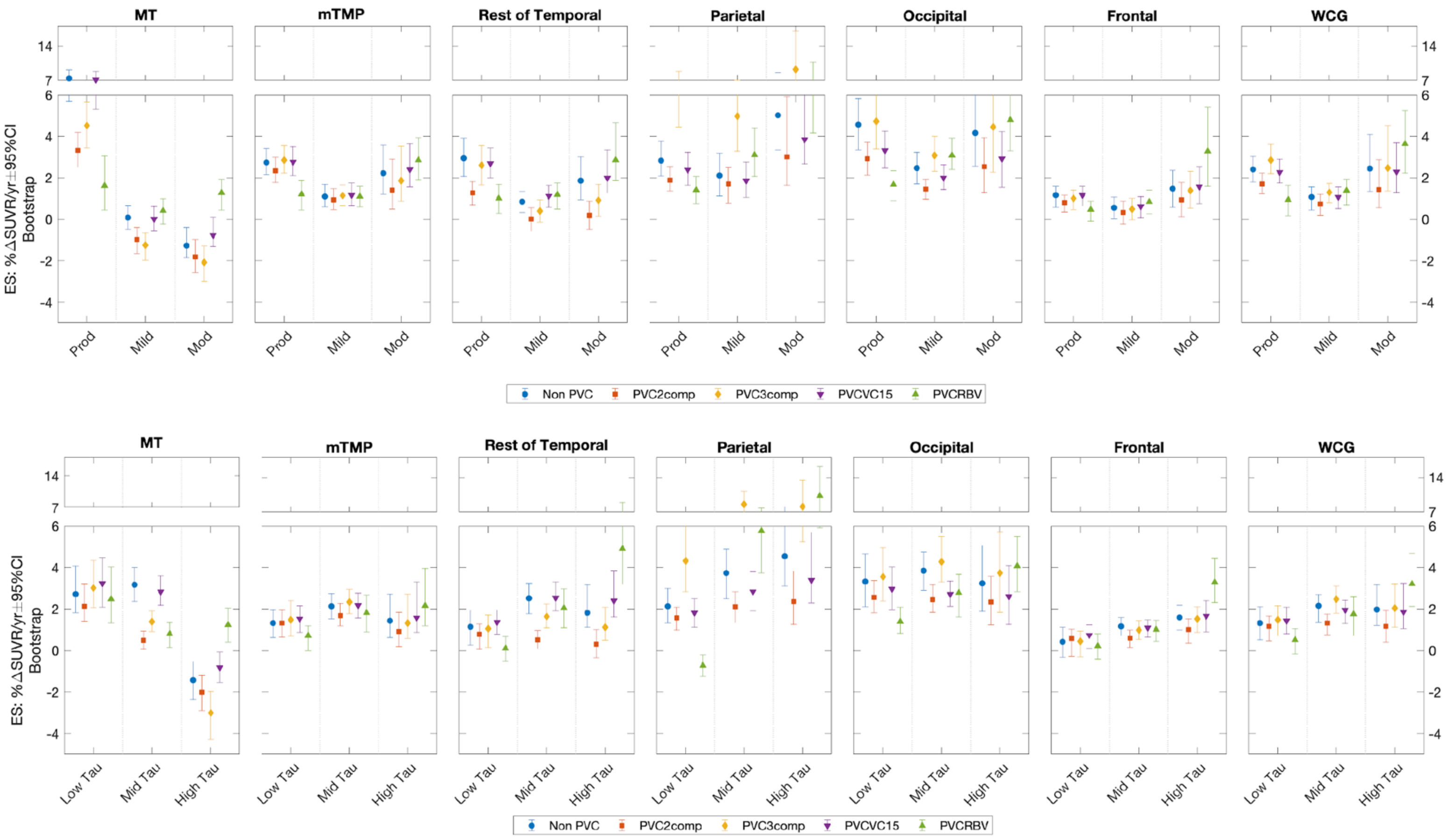

Figure S6. Longitudinal effect size of the average annualized change in SUVR and the corresponding 95% CI in (a) the NHS participants and (b) the Tauriel study measured from the LMEM slope analysis and at the follow-up visits (weeks 52 and 78) relative, baseline. Average and 95% CIs were estimated applying bootstrap.

AD, Alzheimer's disease; CI, confidence interval; CU, cognitive unimpaired; LMEM, linear mixed-effects model; Mild, mild AD; Mod, moderate AD; MT, mesial temporal entorhinal cortex, hippocampus, and amygdala; mTMP, TMP excluding MT; NHS, natural history study; Prod, prodromal AD; SUVR, standardized uptake value ratio; W, week; WCG, whole cortical gray.

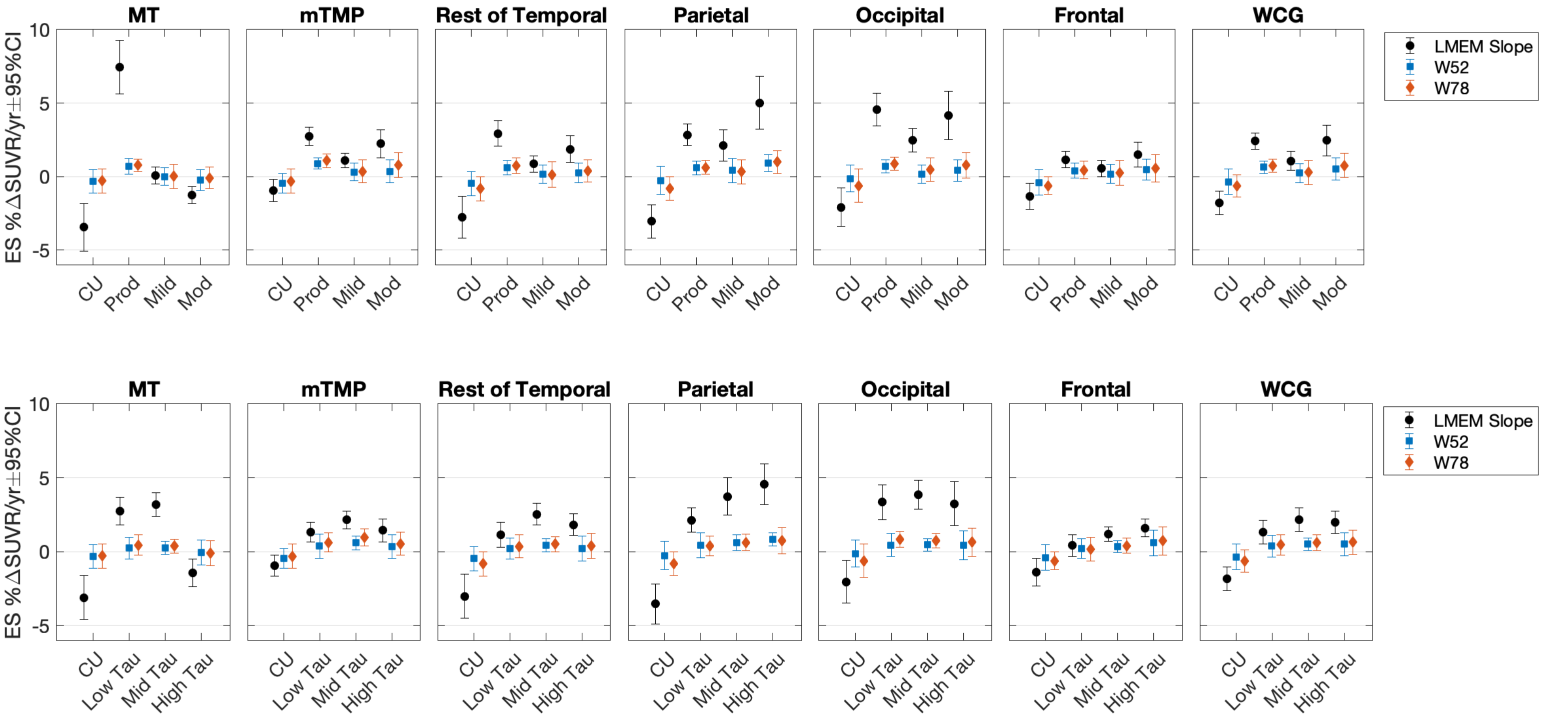

Figure S7. Longitudinal effect size of the average annualized change in SUVR and the corresponding 95% CI in (a) the Tauriel participants and (b) the Tauriel study measured from the LMEM slope analysis and at the follow-up visits (weeks 49 and 73) relative, baseline. Average and 95% CIs were estimated applying bootstrap.

AD, Alzheimer's disease; CI, confidence interval; LMEM, linear mixed-effects model; mAD, mild AD; MT, mesial temporal entorhinal cortex, hippocampus, and amygdala; mTMP, TMP excluding MT; pAD, prodromal AD; SUVR, standardized uptake value ratio; WCG, whole cortical gray; WK, week.

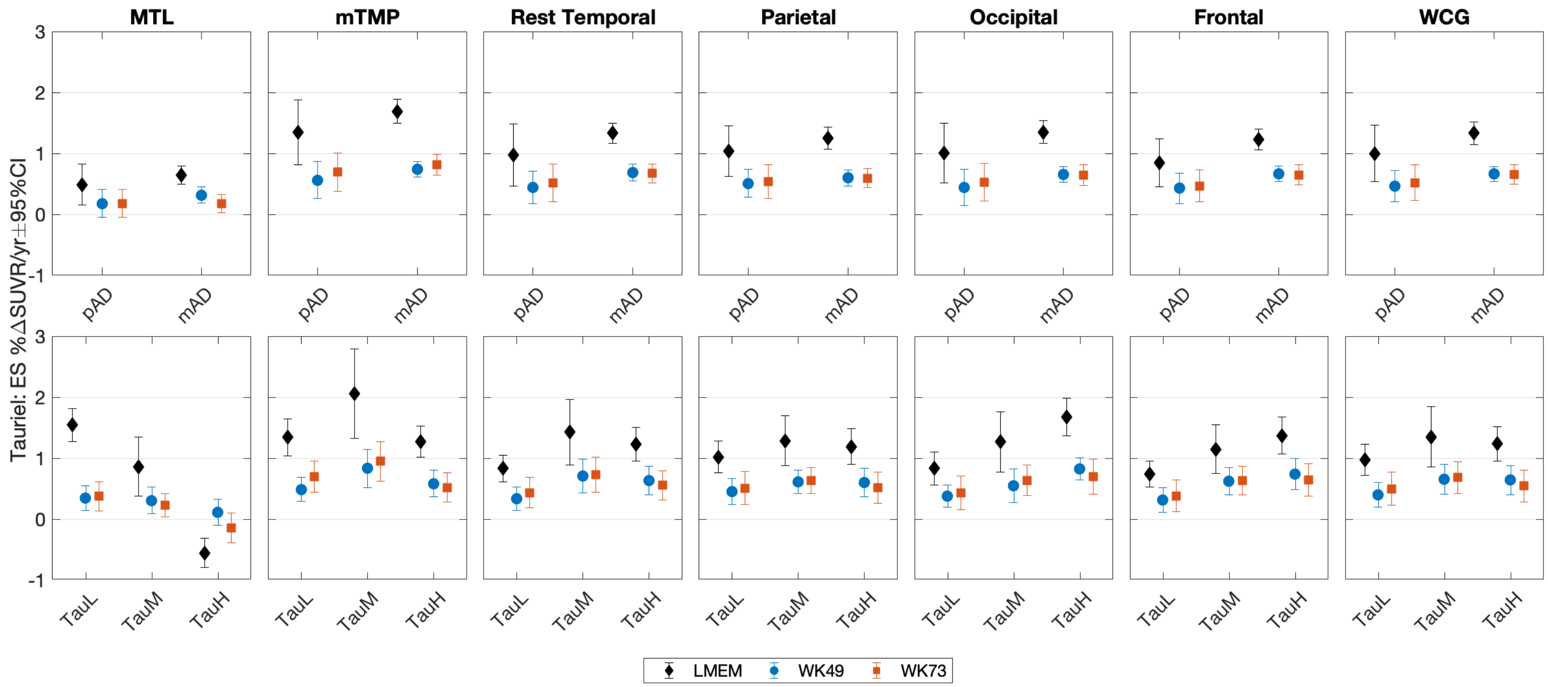

Figure S8. Average annualized change in SUVR and the corresponding 95% CI in the NHS participants estimated from the LMEM slope analysis and at the follow-up visits (weeks 26, 52, and 783) relative, baseline. Subjects are grouped by diagnostic cohort or tau level. Average and 95% confidence intervals were estimated applying bootstrap.

AD, Alzheimer's disease; CU, cognitive unimpaired; LMEM, linear mixed-effects model; Mild, mild AD; Mod, moderate AD; MT, mesial temporal entorhinal cortex, hippocampus, and amygdala; mTMP, TMP excluding MT; Prod, prodromal AD; SUVR, standardized uptake value ratio; W, week; WCG, whole cortical gray.

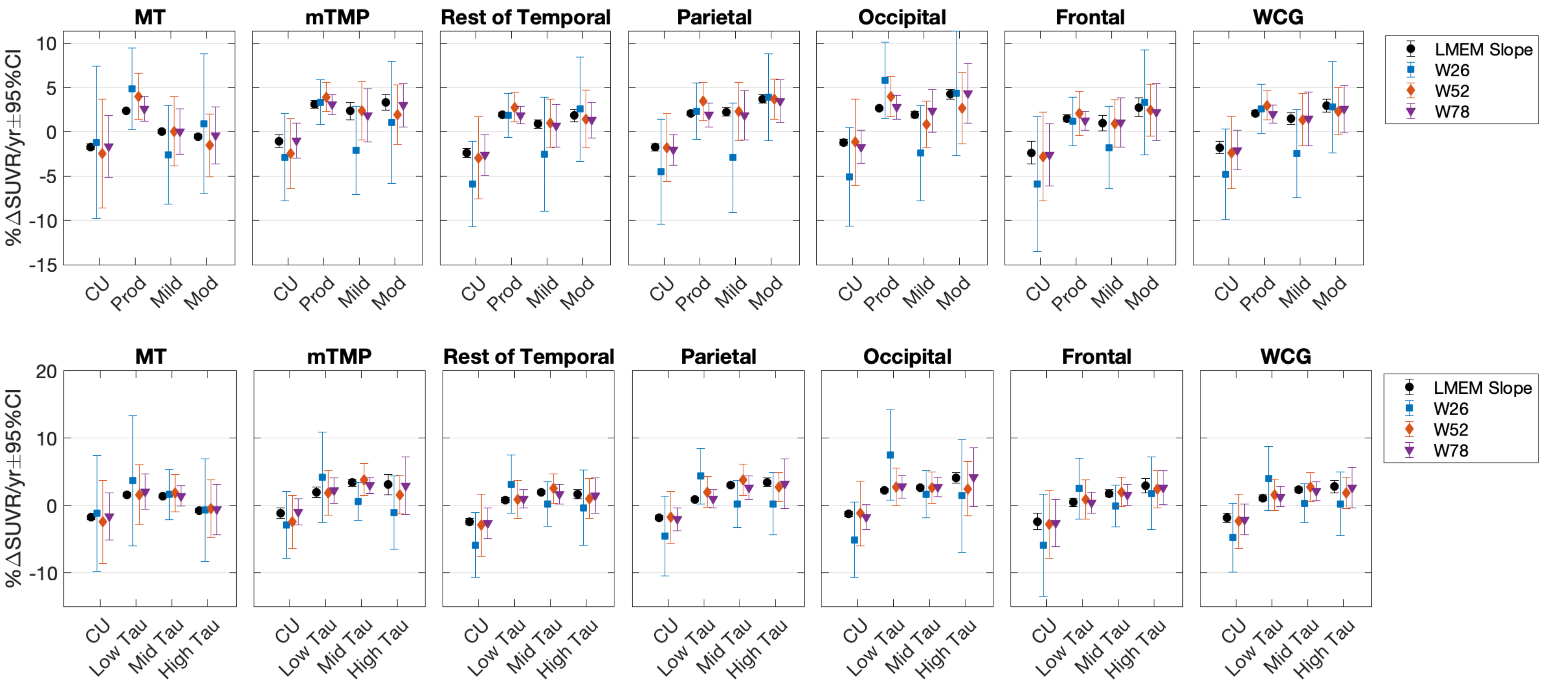

Figure S9. Whole cortical gray [^{18}F]GTP1 SUVR at baseline, and weeks 26, 52, and 78 in the NHS study grouped by diagnostic cohort and by tau level. The participant's SUVR are plotted as a function of their age.

AD, Alzheimer's disease; CU, cognitive unimpaired; Mild, mild AD; Mod, moderate AD; NHS, natural history study; Prod, prodromal AD; SUVR, standardized uptake value ratio.

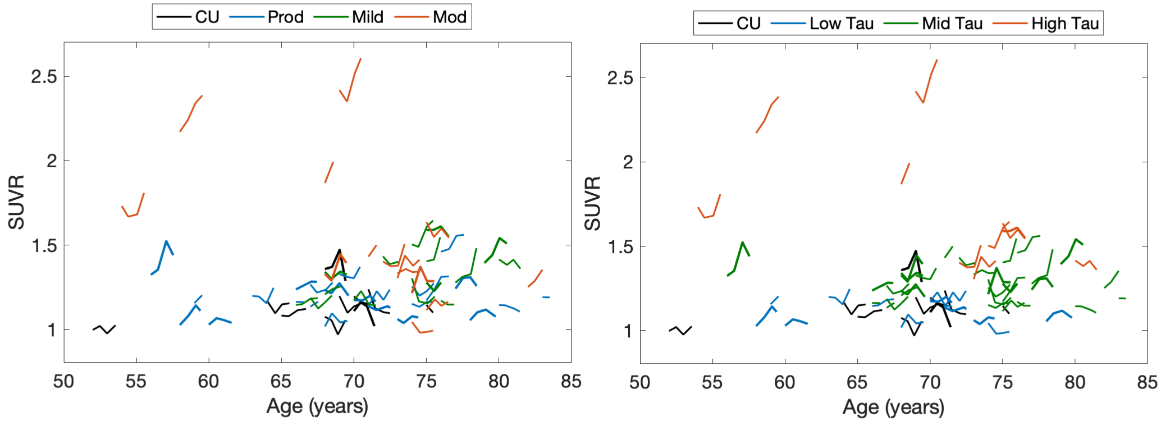

Figure S10. Whole cortical gray [^{18}F]GTP1 SUVR at baseline and weeks 49 and 73 in the Tauriel study grouped by diagnostic cohort and intervention and by tau level. The participant's SUVR are plotted as a function of their age. The percentage of participants displaying a SUVR increase at week 49 followed by decreases at week 73 was about 36% in both placebo and semorinemab arms, 35% and 37% in prodromal and mild AD, respectively, and 28%, 37% and 43% in the low, mid, and high tau groups, respectively, helping, explain the higher annualized SUVR increase at week 49 relative, week 73 and, the LMEM slope analysis.

AD, Alzheimer's disease; LMEM, linear mixed-effects model; SUVR, standardized uptake value ratio.

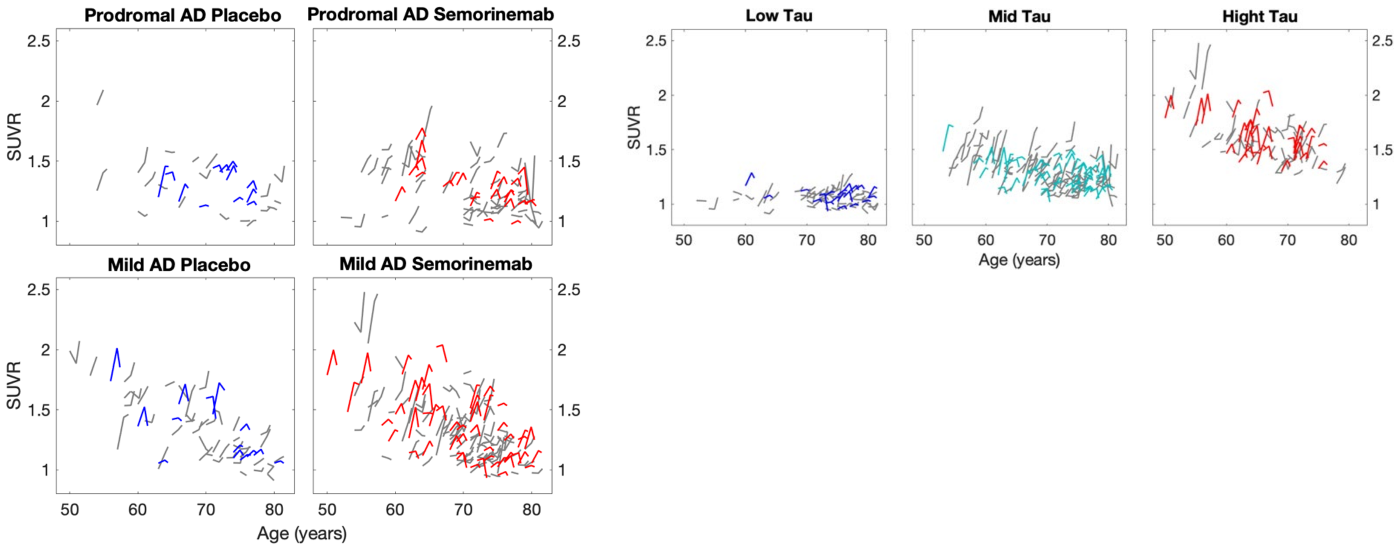

Figure S11. SUVR trajectories in the modified Temporal region (mTMP) for all subjects in the NHS supports the LMEM assumption that the tau burden accumulation can be considered log-linear within a relatively wide SUVR range for the observation period of the study. The residuals distribution does not suggest an under- or over- estimation at the various tau burden levels. Longer observation periods may require, model SUVR using more complex models.

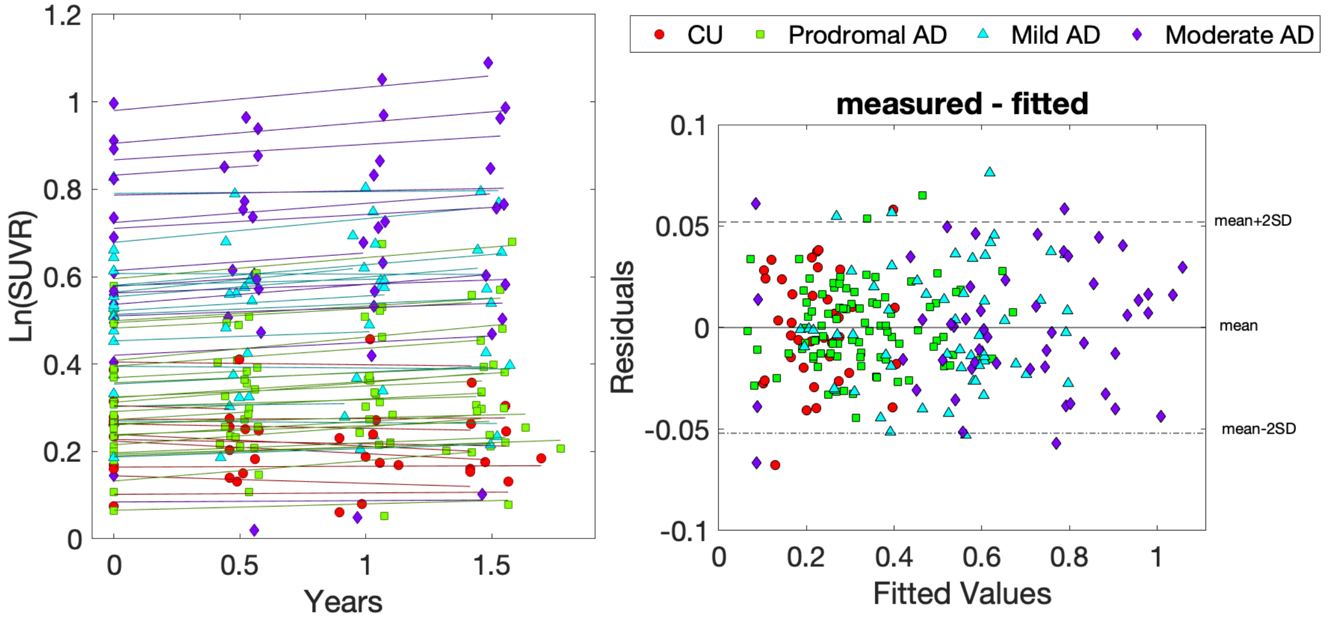

AD, Alzheimer's disease; CU: Cognitive Unimpaired; LMEM, linear mixed-effects model; NHS: Natural History Study; SUVR, standardized uptake value ratio; WCG, whole cortical gray.
